# The oncolytic avian reovirus p17 protein suppresses invadopodia formation via disruption of TKs5 complexes and oncogenic signaling pathways

**DOI:** 10.3389/fcimb.2025.1603124

**Published:** 2025-06-12

**Authors:** Chao-Yu Hsu, Jyun-Yi Li, Wei-Ru Huang, Tsai-Ling Liao, Hsiao-Wei Wen, Chi-Young Wang, Lon-Fye Lye, Brent L. Nielsen, Hung-Jen Liu

**Affiliations:** ^1^ Division of Urology, Department of Surgery, Tungs’ Taichung MetroHarbor Hospital, Taichung, Taiwan; ^2^ Institute of Molecular Biology, National Chung Hsing University, Taichung, Taiwan; ^3^ The iEGG and Animal Biotechnology Center, National Chung Hsing University, Taichung, Taiwan; ^4^ Ph.D Program in Translational Medicine, National Chung Hsing University, Taichung, Taiwan; ^5^ Department of Medical Research, Taichung Veterans General Hospital, Taichung, Taiwan; ^6^ Department of Food Science and Biotechnology, National Chung Hsing University, Taichung, Taiwan; ^7^ Department of Veterinary Medicine, National Chung Hsing University, Taichung, Taiwan; ^8^ Department of Medical Research, Tungs’ Taichung MetroHarbor Hospital, Taichung, Taiwan; ^9^ Department of Microbiology and Molecular Biology, Brigham Young University, Provo, UT, United States; ^10^ Department of Life Sciences, National Chung Hsing University, Taichung, Taiwan; ^11^ Rong Hsing Research Center for Translational Medicine, National Chung Hsing University, Taichung, Taiwan

**Keywords:** oncolytic avian reovirus, p17, p53-PTEN-FAK-Src, TKs5-Nck1 complex, invadopodia formation

## Abstract

**Background:**

Avian reovirus (ARV) is an oncolytic virus that induces autophagy and apoptosis in cancer cells, modulates the immune response, and exposes tumor-associated antigens to the immune system, making it a promising candidate for cancer therapy. Cancer cell migration and invadopodia formation are essential processes in metastasis, and targeting these mechanisms could be beneficial in limiting cancer progression.

**Methods:**

This study investigated the effects of ARV p17 protein on cancer cell migration and invadopodia formation in HeLa and A549 cell lines. Molecular assays were conducted to examine the expression and interactions of key signaling molecules, including nucleoporin Tpr, p53, PTEN, FAK, Src, Rab40b, PI3K, Akt, TKs5, and Nck1. Analysis of TKs5, Nck1, and Rab40b mRNA levels by quantitative real-time RT-PCR. Furthermore, invadopodia detection, gelatin degradation assay, and Fluorescence imaging was performed to visualize invadopodia structures and assess extracellular matrix degradation. Additionally, rescue experiments were performed by co-transfecting cells with mutant PTEN (C124A), TKs5, or Rab40b plasmids to confirm their roles in mediating the effects of p17.

**Results:**

p17 suppressed nucleoporin Tpr, resulting in the activation of p53 and upregulation of PTEN. This blocked the formation of the FAK-Src complex and inhibited the Rab40b-PI3K-Akt signaling pathway. p17 also transcriptionally downregulated TKs5, Nck1, and Rab40b, thereby reducing the formation of TKs5-Nck1 and TKs5-Rab40b complexes, which are critical for invadopodia formation. Fluorescence imaging confirmed a marked reduction in invadopodia formation and matrix degradation in cells expressing p17. Restoration of invadopodia formation upon co-transfection with mutant PTEN, TKs5, or Rab40b confirmed that these molecules are key mediators of p17’s inhibitory effects.

**Conclusion:**

ARV p17 inhibits cancer cell migration and invadopodia formation by activating the p53-PTEN pathway and suppressing essential signaling and scaffolding complexes (FAK-Src, Rab40b-PI3K-Akt, TKs5-Nck1, and TKs5-Rab40b). These findings suggest that p17 plays a crucial anti-metastatic role and may serve as a novel therapeutic agent for targeting invasive cancer cells.

## 1 Introduction

Cancer metastasis, the spread of malignant cells from a primary tumor to distant sites, remains a major challenge in cancer therapy and is often associated with poor prognosis (Cairns et al., 2003; Seyfried and Huysentruyt, 2013). Metastasis involves a complex series of events, including cancer cells’ invasion of surrounding tissues, intravasation into blood or lymphatic vessels, circulation to distant organs, extravasation, and colonization at secondary sites. Among the various mechanisms driving cancer cell invasion and metastasis, the formation of specialized protrusive structures known as invadopodia has emerged as a key factor (Eccles and Welch, 2007; Alonso et al., 2011). Invadopodia are dynamic actin-rich membrane protrusions that facilitate the degradation of extracellular matrix (ECM) components, thereby promoting cancer cell invasion into surrounding tissues (Jacob and Prekeris, 2015; Eddy et al., 2017; Masi et al., 2020). A network of signaling molecules and protein complexes tightly regulates the formation and activity of invadopodia. Regulation of invadopodia is influenced by various signaling pathways, including those activated by receptor tyrosine kinases such as EGFR (Lv et al., 2024), which trigger Src kinase activity and other downstream effectors involved in actin polymerization and invadopodia maturation (Murphy and Courtneidge, 2011; Pelaz and Tabernero, 2022). Additionally, hypoxia and the tumor microenvironment can modulate invadopodia formation by altering the expression of key proteins involved in cytoskeletal dynamics and ECM degradation (Gould and Courtneidge, 2014). Rab40b, a member of the Rab family of small GTPases, has been implicated in various cellular processes, including vesicle trafficking and cytoskeletal dynamics (Linklater et al., 2021). TKs5 (tyrosine kinase substrate with five SH3 domains) is a scaffold protein critical in invadopodia assembly and function (Ala-Aho and Kahari, 2005; Blouw et al., 2015). The interaction between Rab40b and TKs5 facilitates the recruitment of matrix-degrading enzymes and promotes invadopodia-mediated ECM degradation (Jacob and Prekeris, 2015; Cai et al., 2019). The Rab40b-TKs5 complex is necessary for the trafficking and secretion of matrix metalloproteinases (MMPs), such as MMP2 and MMP9, to invadopodia (Jacob et al., 2016; Qi et al., 2020). The TKs5-Rab40b complex is essential for stabilizing invadopodia formation and maturation. Previous reports suggested that the Rab40b-TKs5 complex is a critical regulator of invadopodia formation and function, facilitating the targeted delivery of MMPs to promote ECM degradation and cancer cell invasion (Jacob et al., 2013, 2016; Qi et al., 2020). Targeting this interaction may have therapeutic potential in preventing metastasis.

Avian reovirus (ARV), a member of the Reoviridae family, has been studied extensively for its oncolytic properties and potential therapeutic applications in cancer treatment (Huang et al., 2015; Chiu et al., 2018; Cai et al., 2019; Manocha et al., 2021; Hsu et al., 2022; Huang et al., 2022; Hsu et al., 2024; Wu et al., 2024). These effects have been observed across a variety of human and animal cancer cell lines, where p17 induces autophagy and cell cycle arrest (Chi et al., 2013; Chiu et al., 2016, 2018; Li et al., 2022; Hsu et al., 2022, 2023). Despite these advances, the precise mechanisms by which p17 regulates cancer cell invasion and metastasis remain insufficiently understood. Emerging evidences suggest that specific viral proteins, such as p17, interact with host cell proteins and modulate cellular processes relevant to cancer progression. In this work, we have unveiled a novel role for the ARV p17 protein in suppressing the TKs5-Nck1 and TKs5-Rab40b complexes, key regulators of invadopodia formation. We provide novel insights into the p17-modulated suppression of invadopodia formation by activation of the p53-PTEN pathway to inhibit the FAK-Src complex and by downregulating TKs5, Nck1, Rab40b, and MMP9. Importantly, ARV p17 protein functions as a negative regulator of TKs5-Nck1 and TKs5-Rab40b complexes by activating the p53-PTEN pathway and by suppressing FAK-Src and Rab40b-PI3K-Akt pathways, which inhibits invadopodia formation in HeLa and A549 cancer cells. Finally, we highlight the potential implications of this novel regulatory mechanism for the development of targeted therapies aimed at inhibiting cancer metastasis.

## 2 Materials and methods

### 2.1 Cells and viruses

The S1133 strain of ARV was used in this study. The human cervical cancer (HeLa) cells and adenocarcinomic human alveolar basal epithelial (A549) cancer cell lines are in a mixture containing 10% fetal bovine serum (FBS), 1% penicillin/streptomycin, and 10 mM 4-(2-hydroxyethyl)-1-piperazineethanesulfonic acid (HEPES) (pH 7.2 or 7.0) in Modified Eagle medium (MEM) or Ham’s F-12K (Kaighn’s) medium in a humidified incubator with 5% CO_2_ at 37°C.

### 2.2 Reagents and antibodies

The p17 polyclonal antibodies were produced by our laboratory (Chiu et al., 2018). The Akt III inhibitor, an Akt-specific inhibitor, was purchased from Enzo Life Science (New York, USA). The examination of whether the reduction in TKs5, Nck1, and Rab40b levels, regulated by p17, is mediated through the ubiquitin-proteasome degradation pathway involved transfecting cells with pCI-neo-p17 plasmid DNA for 6 hours, followed by treatment with MG132 at a concentration of 1 mM. MG132 was obtained from Calbiochem Co. (San Diego, USA). Gelatin and Alexa-Fluor-gelatin were purchased from Cell Signaling (Danvers, USA). Anti-mouse IgG (H+L) and anti-rabbit IgG (H+L) antibodies were purchased from KPL (Washington, USA). The catalog numbers and dilution factors of the respective antibodies used in this study are shown in **Table 1**.

**Table 1:** The catalog numbers and dilution factor of the respective antibodies used in this study.

Antibodies	Catalog numbers	Clone name	Dilution factor	Manufacture
Mouse anti-p17*	–	–	2000	Our laboratory (Chiu et al., 2018)
Tpr	sc-100282	L-17	1000	Santa Cruz
Mouse anti-p53	2527	7F5	3000	Cell Signaling
Rabbit anti-p-p53 (S15)*	9284	–	2000	Cell Signaling
Rabbit anti-PTEN	9559	138G6	3000	Cell Signaling
Rabbit anti-p-PTEN (S380/T382/383)	9554	–	2000	Cell Signaling
Rabbit-anti-p-FAK (Y397)	8556		1500	Cell Signaling
Rabbit-anti-FAK	3285		3000	Cell Signaling
Rabbit-anti-p-Src (Y416)	6943		2000	Cell Signaling
Mouse-anti-Src	2110		3000	Cell Signaling
Mouse-anti-Csk			2000	BD
Rabbit-anti-TKs5	16619		3000	Cell Signaling
Rabbit-anti-Rab40b	ab82896		2000	Abcam
Rabbit-anti-Nck1	2319		3000	Cell Signaling
Rabbit-anti-MMP9	13667		3000	Cell Signaling
Mouse anti-cortactin	3503		3000	Cell Signaling
Alexa Fluor 568 Phalloidin	13054		500	Cell Signaling
Mouse anti-β-actin	MAB1501	C4	10000	Millipore
Rabbit anti-p-Tyr-1000	8954		2000	Cell Signaling
Goat anti-mouse IgG (H+L) HRP	5220-0341	–	5000	SeraCare
Goat anti-rabbit IgG (H+L) HRP	5220-0336	–	5000	SeraCare

*Polyclonal antibodies.

### 2.3 shRNAs and transient transfection

The pLKO-AS1-puro plasmid encoding the shRNA was obtained from the National RNAi Core Facility of Academia Sinica, Taiwan. The shRNA sequences of this experiment are shown in **Supplementary Table S1**. In this work, HeLa and A549 cells were transfected or co-transfected with the respective shRNAs or plasmids for 24 h. The scramble plasmid was used as a negative control. Cell viability measurements with the MTT assay were conducted following transfections with the respective shRNAs or plasmids. The transfection efficiency was confirmed either by Western blot or immunofluorescence staining to ensure that the transfection efficiency reached 80-90%. In knockdown assays, cells were first transfected with the respective shRNA for 6 hours, followed by transfection of the respective vectors or infection with ARV at MOI of 10 for 24 hours. Whole-cell lysates were collected for Western blot analysis.

### 2.4 Reverse transcription and polymerase chain reaction and plasmid construction

To prepare cDNA for Rab40b and TKs5 genes, total RNA was extracted from Vero cells using TRIzol solution (Thermo Fisher Scientific Inc., Waltham, USA) according to the manufacturer’s protocol. The Rab40b and TKs5 gene fragments were amplified by PCR using the specific primers shown in **Supplementary Table S2**. Total RNA was extracted from A549 and HeLa cells using the TRIzol reagent (Thermo Fisher Scientific Inc., Waltham, USA) according to the manufacturer’s procedures. PCR products were subsequently purified and inserted into the pcDNA3.1 vector at the corresponding restriction sites. Reverse transcription was carried out at 42°C for 15 minutes, followed by a 72°C incubation for an additional 15 minutes. The PCR reactions were set up in a 20 μL volume, containing 1 μL of cDNA, 1 μL of each primer, 2 μL of PCR mix, and 15 μL of nuclease-free water. Amplification was initiated with an initial denaturation at 95°C for 5 minutes, followed by 35 cycles of 95°C for 30 seconds, 55°C for 60–90 seconds, and 72°C for 1 minute, with a final extension at 72°C for 10 minutes.

To explore whether p17-modulated suppression of TKs5-NcK1 and TKs5-Rab40b complexes, pDNA3.1-Rab40b and pDNA3.1-TKs5 plasmids were constructed. To further study the involvement of p17 in invadopodia formation in A549 and HeLa cells, we also constructed pcDNA3.1-PTENC124A (a dominant-negative mutant of PTEN). The PTENC124A mutation impairs PTEN’s phosphatase activity, thereby inhibiting its ability to dephosphorylate substrates. The pCI-neo-p17 vector was constructed as described previously (Huang et al., 2022).

### 2.5 Analysis of TKs5, Nck1, and Rab40b mRNA levels by quantitative real-time RT-PCR

To assess the impact of p17 on the transcriptional regulation of TKs5, Nck1, and Rab40b, total RNA was extracted from cells transfected with p17 using the TRIzol reagent. Quantitative real-time RT-PCR was performed using the iQ™ SYBR^®^ Green Supermix kit (Bio-Rad, Hercules, FL, USA) as previously described (Huang et al., 2015, 2022). The GAPDH expression was used as an internal control.

### 2.6 Electrophoresis and Western blot assays

The day before infection with the virus or transfection with plasmids as described above, cells were seeded in 6-well cell culture dishes. The collected cells were washed twice with 1X PBS and lysed with lysis buffer (Cell Signaling, USA). The Bio-Rad Protein Assay (Bio-Rad Laboratories, USA) was used to determine the concentration of solubilized protein in cell lysates according to the manufacturer’s protocol. Samples were mixed with an equal amount of 5X Laemmli loading buffer and boiled in a water bath for 10–20 minutes. Samples were run on 10% or 12% sodium dodecyl sulfate (SDS)-polyacrylamide gels and transferred to PVDF membranes (GE Healthcare Life Sciences, Chicago, USA). Protein expression was detected using the appropriate primary antibody and horseradish peroxidase secondary antibody conjugate and exposed to X-ray film (Kodak, Rochester, USA) after incubation of the membrane with enhanced chemiluminescence (ECL plus) reagent (Amersham Biosciences, Little Chalfont, England). Amounts of target protein were calculated using ImageJ.

### 2.7 Wound healing assays

To assess the effects of p17 on the migration of A549 and HeLa cancer cells, a wound healing assay was conducted using a pipette tip to generate scratches in the cell monolayer (Freitas et al., 2021; Hsu et al., 2024). Twenty-four hours post-transfection with the pcDNA3.1-p17 vector, the migration of the cells was monitored by capturing images of the wound area. Additionally, A549 and HeLa cells were co-transfected with pcDNA3.1-p17 or shRNAs targeting Tpr, p53, PTEN, Rak, or ROCK for 24 hours. As p53, Rak, and ROCK are known to enhance PTEN activity, their expression was silenced using shRNA (Huang et al., 2015). The inhibitory effects of Tpr, p53, PTEN, Rak, and ROCK shRNAs were examined in cells. As described previously (Hsu et al., 2024), cell migration was evaluated by measuring the gap between the scratch edges at 12 and 24 hours post transfection, with images taken at 0, 12, and 24 hours to track progress. After transfecting different shRNAs into cells, samples were collected at 0, 6, 12, 18, and 24 h, respectively. The inhibitory effect was confirmed by Western blotting. Additionally, the GFP of pGFP-V-RS vector was expressed, and the transfection efficiency can be confirmed by fluorescence.

### 2.8 Co-immunoprecipitation assays

To analyze the interactions between FAK-Src, TKs5-Nck1, and TKs5-Rab40b, co-immunoprecipitation assays were conducted using the Catch and Release Reversible Immunoprecipitation System (Millipore, Merck Ltd., Taipei, Taiwan) as described previously (Chiu et al., 2018; Huang et al., 2022). A549 and HeLa cells were seeded in 6-cm dishes and cultured in MEM or F-12K medium supplemented with 5% FBS, then incubated at 37°C with 5% CO_2_ until approximately 75% confluence was reached. Following transfection with the appropriate plasmids, cells were harvested 24 hours post transfection. At this point, the cells were washed twice with 1× PBS, scraped, and lysed in 200 μL of CHAPS lysis buffer (40 mM HEPES [pH 7.5], 120 mM NaCl, 1 mM EDTA, 10 mM pyrophosphate, 10 mM glycerophosphate, 50 mM NaF, and 0.3% CHAPS). To perform the immunoprecipitation, 1000 μg of protein from each sample was incubated overnight at 4°C with 4 μg of the corresponding antibody. The immunoprecipitated protein complexes were resolved by SDS-PAGE and analyzed through Western blotting using the specified antibodies. Rabbit IgG was included as a negative control to confirm specificity.

### 2.9 Invadopodia detection and gelatin degradation assay

Invadopodia, which are primarily composed of *β*-actin and cortactin, are essential for cancer cell migration. These structures enable cells to degrade the extracellular matrix, contributing to cancer cell invasion. When cell migration is inhibited, the formation of invadopodia is suppressed, as *β*-actin and cortactin fail to aggregate. To determine if the ARV p17 can inhibit invadopodia formation, cancer cells were either mock-transfected or co-transfected with p17, TKs5, Rab40b, or corresponding shRNAs for 24 hours. After transfection, cells were washed with PBS and fixed in 4% formaldehyde. Primary antibodies targeting *β*-actin and cortactin were applied overnight at 4°C. Following incubation, cells were washed with PBST containing Triton X-100 and incubated with fluorescently labeled secondary antibodies (1:500 dilution) for 2 hours at room temperature. After five additional washes with PBST, cells were stained with DAPI (Vector Laboratories, USA) and analyzed using a confocal microscope (Olympus FV1000, Tokyo, Japan).

Invadopodia are dynamic, actin-rich protrusions crucial for cancer cell attachment to and degradation of ECM, playing an essential role in tumor metastasis (Sells et al., 1999; Bowden et al., 2001; Van Horssen et al., 2013). To investigate whether ARV infection affects invadopodia formation, a gelatin degradation assay was performed. Fluorophore-conjugated gelatin-coated coverslips were prepared to simulate the ECM. First, the coverslips were washed with 1N hydrochloric acid for 12–16 hours, followed by water rinsing and sterilization with 70% ethanol. The coverslips were then incubated with 50 μg/mL poly-L-lysine (Merck Ltd., Taipei, Taiwan) at room temperature for 20 minutes. After two washes with PBS, the coverslips were fixed in ice-cold 0.5% glutaraldehyde for 15 minutes and subsequently washed with PBS. The coverslips were placed upside down on an 80 μL droplet of fluorescent gelatin matrix (0.2% gelatin and Alexa-Fluor-gelatin, 8:1) and incubated for 15 minutes at room temperature. Following two PBS washes, the matrix was reduced by incubating in PBS containing 5 mg/mL sodium borohydride for 10 minutes, followed by two PBS washes. In transfection and knockdown assays, A549 and HeLa cells were seeded onto the gelatin-coated coverslips and allowed to reach approximately 70% confluence. The cells were then co-transfected with p17 and the respective plasmids or shRNAs for 24 hours. After transfection, the cells were washed with PBS and fixed in 4% formaldehyde at room temperature for 20 minutes. Following fixation, the cells were washed with PBS and permeabilized with a blocking buffer containing 0.1-0.3% Triton X-100 for 10–20 minutes. After washing with PBS to remove excess detergent, cells were immunostained overnight at 4°C with *β*-actin primary antibody (DyLight 554 Phalloidin). Finally, cells were examined using a confocal microscope (Olympus FV1000, Tokyo, Japan) to assess *β*-actin and to evaluate whether the fluorescent gelatin matrix was degraded by invadopodia. The areas where the matrix had been degraded, leaving behind regions without fluorescence, were recorded and measured.

### 2.10 Statistical analysis

The data, collected from three independent experiments, are presented as the mean ± standard error (SE). The statistical significance of the results was analyzed using Duncan’s Multiple Range Test (MDRT) with Prism 8 software (GraphPad, San Diego, USA). Similar letters (a, b, c) indicate no significant difference.

## 3 Results

### 3.1 The ARV p17 protein downregulates nucleoporin Tpr and activates the p53-PTEN pathway in HeLa and A549 cancer cells

**Figure 1:**
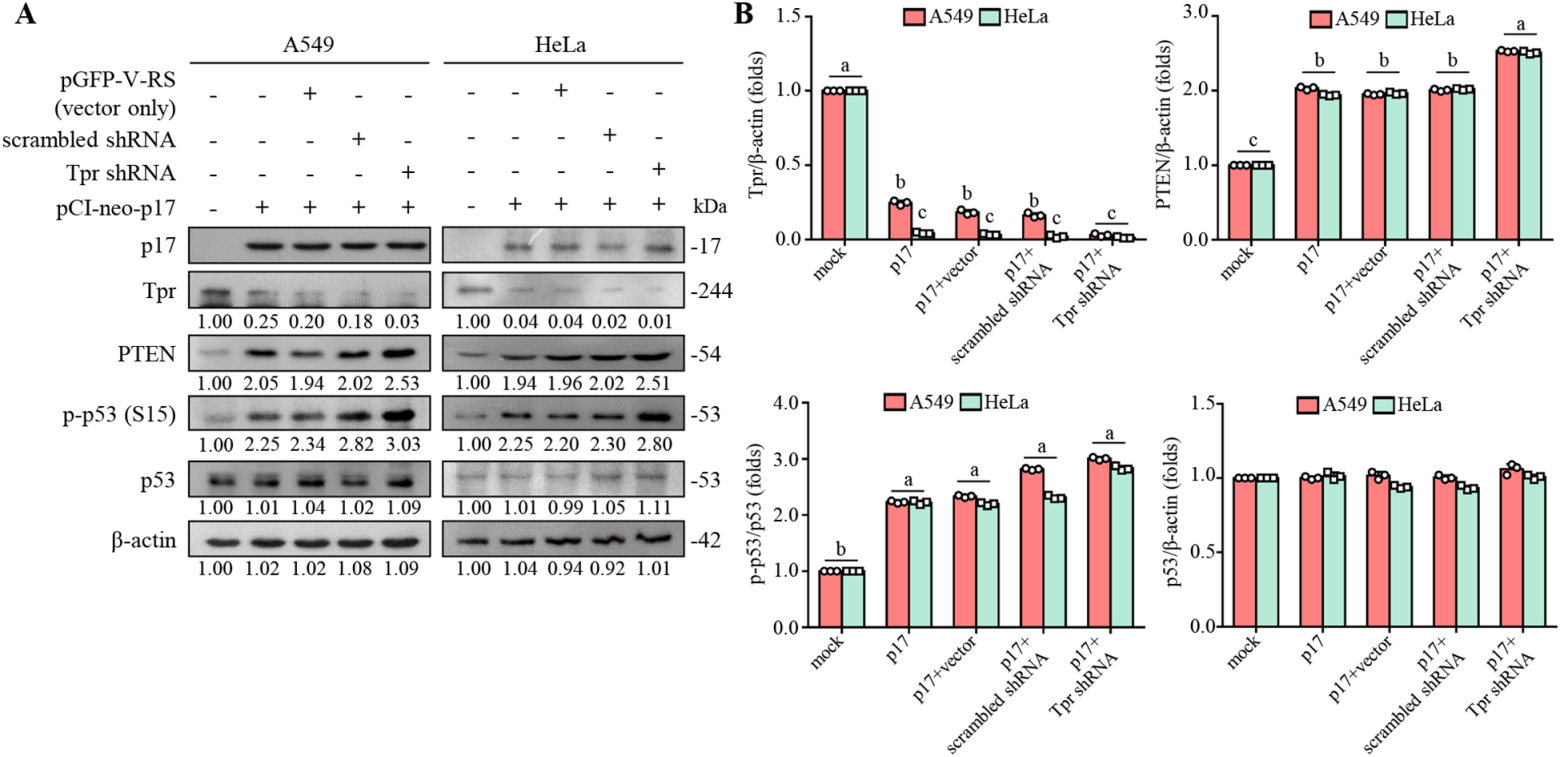
The ARV p17 protein functions as a Tpr suppressor, leading to the activation of p53 and PTEN in various cancer cell lines. **(A)** The expression levels of Tpr, PTEN, phosphorylated form of p53 (p-p53), and p53 were analyzed in cancer cells transfected with either pcDNA3.1 or p17, as well as in untreated control cells. Whole-cell lysates were collected 24 hours post-transfection, and protein levels were examined via Western blot assays. β-actin was used as a loading control. The fold changes in activation and inactivation indicated below each lane, were normalized against those in the mock-transfected cells. Protein expression levels in the mock group were set as 1-fold for comparison. **(B)** Signals for all blots were quantified using ImageJ software. Data in panel A are means and SE from three independent experiments. All original blots and images are shown in **Supplementary Figure S4**.

Our previous studies confirmed that p17 inhibits nucleoporin Tpr, thereby promoting the accumulation of p53 in the nucleus and further activating p53, PTEN, and p21 in Vero cells (Huang et al., 2015; Chiu et al., 2018). To confirm whether p17 leads to similar phenomena in A549 and HeLa cancer cells, Western blot assays were performed to analyze signal changes in cells transfected with the pCI-neo-p17 plasmid or co-transfected with the Tpr shRNA. Our results showed that p17 significantly reduced the expression of nucleoporin Tpr by approximately 70% in both A549 and HeLa cells (*p*<0.01), accompanied by a 1.8-fold increase in PTEN levels and a 2.2-fold increase in phosphorylated p53 (Ser15) (*p*<0.01; **Figures 1A, B**). Notably, co-transfection of pCI-neo-p17 with Tpr shRNA further enhanced this effect, resulting in a 2.6-fold increase in PTEN and a 3.1-fold increase in p-p53 (S15) levels compared to the control group (*p*<0.01; **Figures 1A, B**).

### 3.2 The p17 protein effectively inhibits the migration of A549 and HeLa cancer cells

To investigate whether p17 can inhibit the migration of A549 and HeLa cancer cells, we conducted wound healing experiments to study the migration of cancer cells (Fares et al., 2020; Freitas et al., 2021). This assay was used to test and record the migration distance of A549 and HeLa cancer cells at 12- and 24-hour post-transfection. Our results indicate that p17 effectively inhibits the migration of both A549 and HeLa cancer cells compared to the control group. Given our previous findings that p17 positively regulates p53 and Rak, and drives the translocation of *β*-arrestin-mediated PTEN from the cytoplasm to the plasma membrane in a ROCK-1-dependent manner (Huang et al., 2015; Li et al., 2019), we utilized shRNA to deplete p53, PTEN, Rak, and ROCK-1. Our findings demonstrate that depletion of p53, PTEN, Rak, and ROCK-1 reversed the p17-mediated inhibition of cell migration (**Supplementary Figures S1A–C**), suggesting that p17 inhibits cell migration through a p53-PTEN-dependent pathway. In contrast, Tpr depletion did not significantly alter the p17-mediated inhibition of cell migration (**Supplementary Figures S1A–C**).

### 3.3 The ARV p17 protein enhances PTEN expression in HeLa and A549 cells and inhibits the phosphorylation of Y397 on focal adhesion kinase

**Figure 2:**
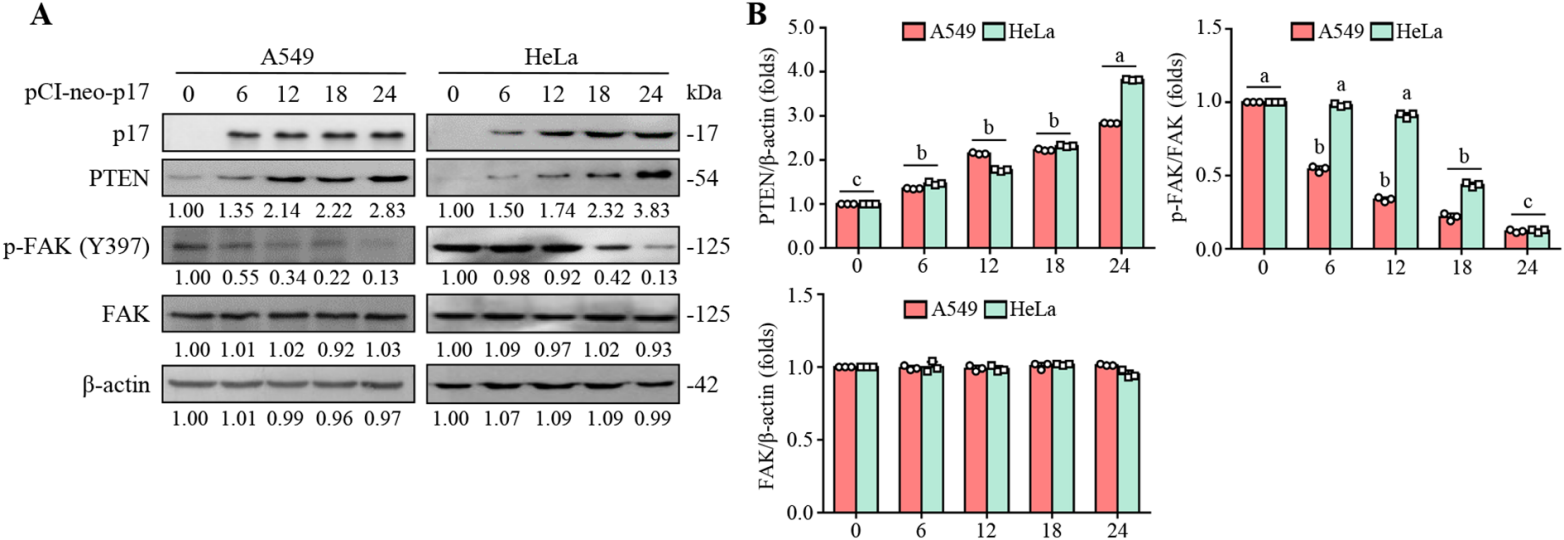
The ARV p17 protein promotes PTEN expression in HeLa and A549 cells and inhibits FAK phosphorylation at Y397. **(A)** The expression levels of PTEN, phosphorylated FAK (p-FAK Y397), and total FAK were analyzed in p17-transfected cancer cells and untreated control cells. Whole-cell lysates were collected at 0, 6, 12, 18, and 24 hours post-transfection, followed by Western blot analysis. β-actin was used as a loading control. The fold changes in activation and inactivation indicated below each lane, were normalized against those in the mock-transfected cells. Protein levels in the mock group were set as 1-fold for comparison. **(B)** Signals for all blots were quantified using ImageJ software. Data in panel A are means and SE from three independent experiments.

**Figure 3:**
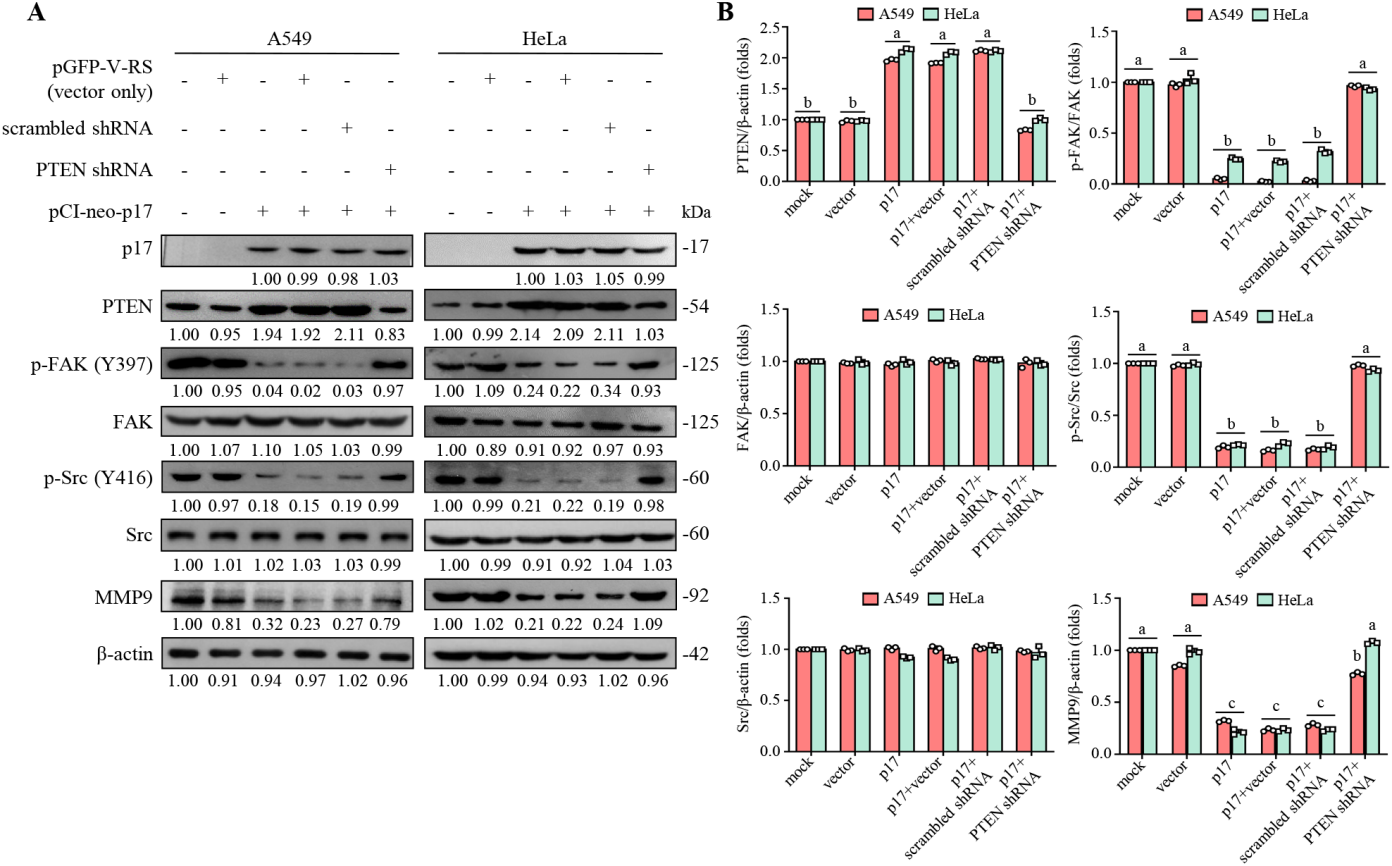
The p17 protein of ARV upregulates PTEN to inhibit the FAK/Src/MMP9 pathway. **(A, B)** The expression levels of PTEN, p-FAK, FAK, p-Src, Src, and MMP9 in pcDNA3.1-transfected, and p17-transfected cancer cells, and cells only were examined. Whole-cell lysates were collected at 24 hr post-transfection for Western blot assays. β-actin was included as a loading control. The fold activation and inactivation indicated below each lane were normalized against those at mock. The levels of indicated proteins at mock treatment were considered 1-fold. **(B)** Signals for all blots were quantified using ImageJ software. Data in panel A are means and SE from three independent experiments.

FAK is essential for regulating integrin-mediated cell adhesion and cancer cell migration (Chan et al., 2009). Consequently, we investigated the effect of the ARV p17 protein on FAK signaling. As shown in **Figure 2**, the pCI-neo-p17-transfected cells exhibited increased PTEN levels accompanied by a decreased level of p-FAK (Y397) compared to HeLa and A549 cancer cells alone. We next explored whether the dephosphorylation of FAK by p17 is dependent on PTEN. Previous studies indicate that FAK is phosphorylated at Tyr 397, creating a crucial binding site for Src family kinases, the p85 regulatory subunit of phosphatidylinositol 3-kinase (Chen and Guan, 1994), and phospholipase Cγ (Zhang et al., 1999). Notably, Src binding to Tyr 397 is necessary for subsequent phosphorylation at Y576/Y577, which is critical for the full activation of FAK. Once activated, the FAK-Src complex phosphorylates multiple adhesion components involved in the dynamic regulation of cell motility and invadopodia formation. In this study, p17 significantly reduced the phosphorylation of FAK (Y397) and Src (Y416), as well as MMP9 protein levels. p-FAK (Y397) was reduced by 85%, p-Src (Y416) by 80%, and MMP9 by 70% in A549 and HeLa cells. Importantly, these inhibitory effects were reversed in PTEN knockdown cells (all *p*<0.01; **Figures 2A, B**, **Figures 3A, B**), where p-FAK (Y397), p-Src (Y416), and MMP9 levels were restored to near-control levels (within 90-100% of baseline, *p*>0.05). These findings indicate that p17 suppresses the FAK-Src pathway and reduces MMP9 expression in a PTEN-dependent manner.

### 3.4 The ARV p17 protein effectively inhibits the complex formation of FAK-Src in A549 and HeLa cancer cells

**Figure 4:**
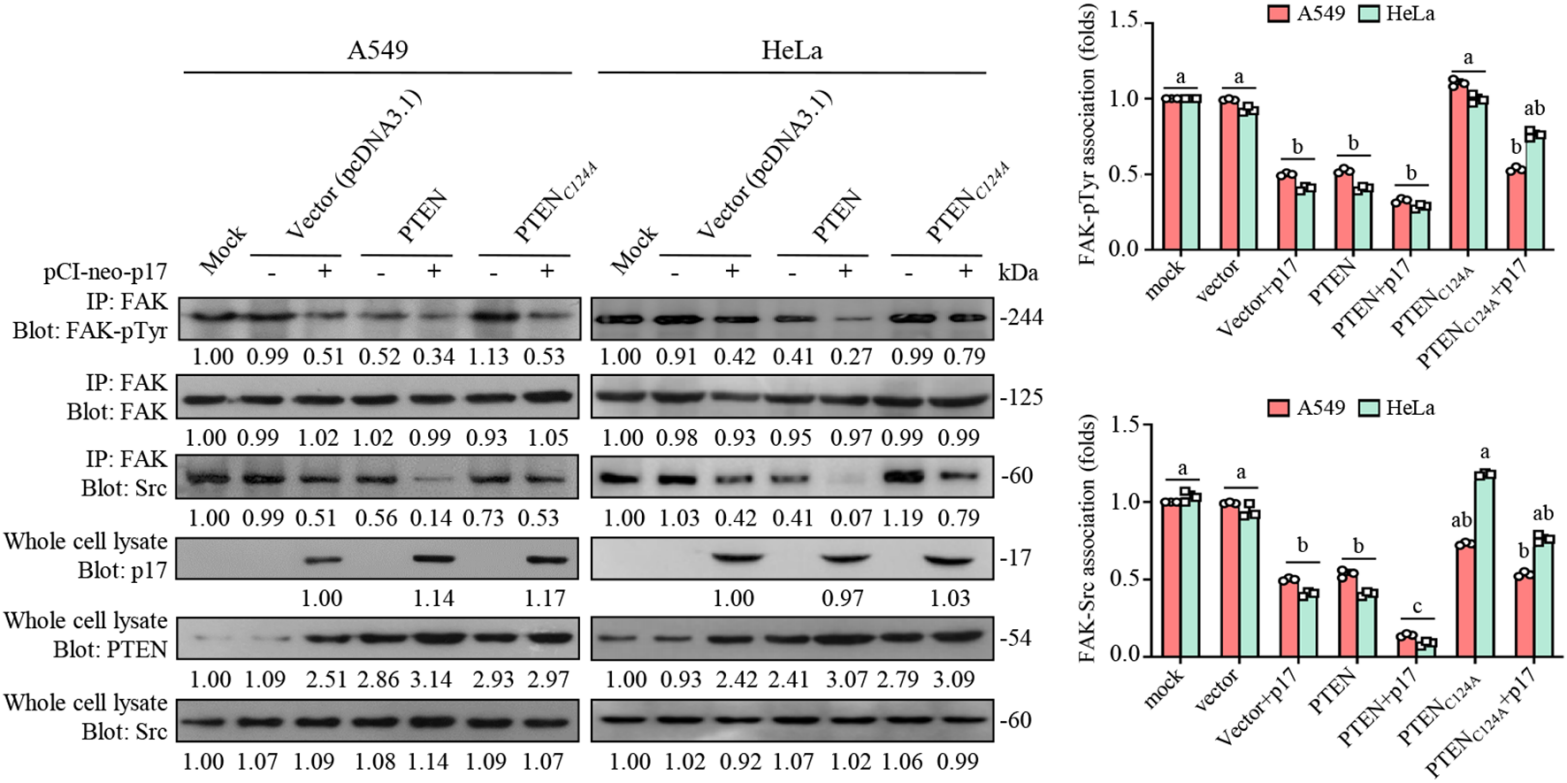
The ARV p17 protein activates PTEN, leading to the inhibition of FAK/Src complex formation. Co-immunoprecipitation experiments were performed to assess the interaction between FAK, phosphorylated FAK (FAK-pTyr), and Src in cancer cells. The experiments were carried out as described previously. The levels of the indicated proteins in control cells or cells subjected to mock transfection were used as the baseline and considered 1-fold for comparison. The results demonstrate that activation of PTEN by p17 disrupts the formation of the FAK/Src complex, contributing to the inhibition of downstream signaling pathways that promote cell migration. Signals for all blots were quantified using ImageJ software. Immunoblots are means and SE from three independent experiments.

Previous reports have shown that the major autophosphorylation site of FAK (Y397) is responsible for the initial *in vivo* association of PTEN with FAK, which is a prerequisite for PTEN to dephosphorylate FAK (Tamura et al., 1999a). PTEN interacts with FAK and reduces its tyrosine phosphorylation at Tyr 397 (Tamura et al., 1998), preventing Src from binding to FAK. Therefore, we next investigated the molecular interactions of FAK and Src in A549 and HeLa cancer cells. In our investigation of the molecular interactions between FAK and Src in A549 and HeLa cancer cells, we observed that the p17 protein significantly reduced the phosphorylation of FAK at Tyr397 and the phosphorylation of Src, leading to a decrease in the formation of the FAK-Src complex (**Figure 3A**). However, the inhibition of complex formation was partial, as evidenced by the co-immunoprecipitation assays. Specifically, the overexpression of p17 reduced FAK phosphorylation and inhibited the formation of the FAK-Src complex, and this effect was further strengthened by the overexpression of PTEN. In contrast, overexpression of the PTEN C124A mutant, which lacks lipid phosphatase activity, did not significantly reduce FAK-Src complex formation compared to the control group (1.02± 0.08-fold; n= 3). However, co-transfection of p17 and PTEN C124A led to a partial restoration of the inhibitory effect, reducing FAK-Src complex formation by approximately 28% relative to PTEN C124A alone (0.72 ± 0.05-fold; p <0.05; **Figure 4**). These findings suggest that p17-mediated the PTEN pathway, leading to FAK dephosphorylation at Tyr397, blocks Src binding and disrupts Src complex formation. The reduction in FAK phosphorylation at Tyr397 has significant biological implications (Tamura et al., 1998). This phosphorylation site is critical for the recruitment of Src to FAK, which is a key step in activating downstream signaling pathways that regulate cell migration, invasion, and survival. By inhibiting this phosphorylation, p17 reduces the recruitment of Src to the FAK complex, leading to impaired activation of the FAK-Src signaling axis. As a result, cancer cell processes such as migration, invasion, and potentially survival are suppressed. Moreover, Src inhibition could lead to a reduction in integrin signaling, further contributing to the inhibition of cancer cell migration and invasion. Thus, the p17 protein appears to act as a potent regulator of the FAK-Src axis by activating PTEN, which in turn dephosphorylates FAK, ultimately impairing cancer cell motility and invasion.

### 3.5 The p17 protein suppresses TKs5 phosphorylation through suppression of Src activity in cancer cells

**Figure 5:**
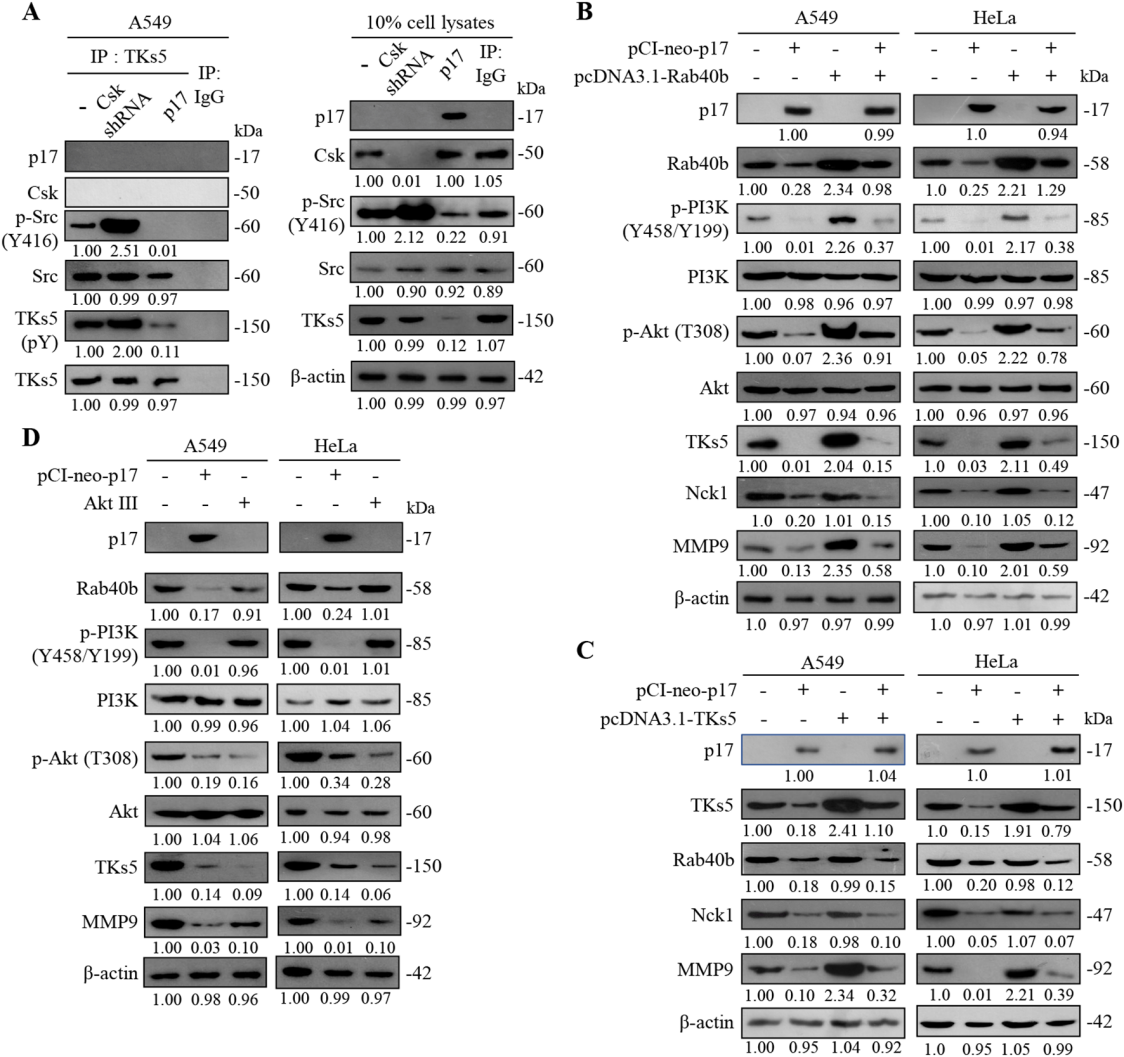
The ARV p17 protein inhibits the expression levels of TKs5, Rab40b, NcK1, and downstream MMP9. **(A)** To determine whether p17 modulates the formation of the Src-TKs5 complex, a co-immunoprecipitation assay was conducted in p17-transfected cells and Csk shRNA-transfected cells. The assay aimed to assess the interaction between Src and TKs5 in the presence of p17. **(B, C)** A549 cells were co-transfected with pCI-neo-p17 and pcDNA3.1-Rab40b and pcDNA3.1-TKs5. After 24 hours of transfection, cell lysates were collected and analyzed by Western blotting. The protein expression levels in the mock-transfected group were used as the baseline and considered 1-fold for comparison. **(D)** The expression levels of TKs5 and MMP9 were further analyzed in p17-transfected cells and cells treated with the Akt inhibitor, Akt III. The results reinforced that p17 inhibits TKs5 and MMP9 expression, confirming its role in suppressing the TKs5/Rab40b pathway and downstream MMP9 activity. Signals for all blots were quantified quantitated by densitometric analysis using ImageJ software (**Supplementary Figure S2**). Data in panels **(A–D)** are means and SE from three independent experiments.

As described in previous reports, TKs4 and TKs5 are known Src substrates that play important roles as organizers of invadopodia (Diaz et al., 2009; Saykali and El-Sibai, 2014; Blouw et al., 2015). A previous study has shown that Src phosphorylates TKs5, a modification that enhances cell migration (Burger et al., 2014). This work further validates that the ARV p17 protein enhances PTEN-mediated inhibition of FAK phosphorylation and suppression of Src. To investigate whether p17 suppresses TKs5 phosphorylation by inhibition of Src, Csk shRNA was used to increase Src activity, aiming to provide supporting evidence for p17’s regulatory effect. The results showed that in the Csk shRNA group, where Src activity was enhanced, phosphorylation of Src at Y416 increased by approximately 2.5-fold compared to the control group (p< 0.01). Correspondingly, TKS5 phosphorylation was elevated by 20-fold relative to the pCI-neo-p17-transfected group (**Figure 5A**). These observations strongly suggest that “the p17 protein suppresses TKs5 phosphorylation in cancer cells through suppression of Src activity.” By modulating this pathway, p17 effectively limits TKs5 activation, which is crucial for invadopodia formation and cell migration. As such, the inhibitory effect of p17 on Src-mediated TKs5 phosphorylation highlights its potential as a key regulator in reducing cancer cell motility, providing insights into its therapeutic implications in the suppression of cancer metastasis.

### 3.6 The p17 protein suppresses TKs5-Rab40b complex formation

The formation of invasive pseudopodia is closely related to the activity of PI3K (Phosphoinositide 3-kinase) (Yamaguchi et al., 2011; Hoshino et al., 2013; Xue and Hemmings, 2013). PI3K plays a crucial role in cell motility, invasion, and metastasis, particularly in the behavior of tumor cells. By activating downstream signaling pathways, PI3K promotes cell survival and proliferation. A previous report has shown that the activity of PI3K is closely linked to the reorganization of the cytoskeleton, especially during the process of pseudopodia formation (Cantley, 2002). Members of the Wiskott-Aldrich syndrome protein (WASP) family, such as WAVE1 and WAVE2, can enhance actin polymerization by activating the PI3K signaling pathway, thereby increasing the formation of pseudopodia and the migratory ability of cells (Oser et al., 2009). It has been confirmed that the ARV p17 protein can inhibit the phosphorylation of TKs5, which affects the TKs5-Rab40b complex formation. In this work, by incrementally expressing TKs5 and Rab40b proteins, it was demonstrated that the transfection of the pCI-neo-p17 resulted in a downward trend in TKs5, Rab40b, and the downstream MMP9 compared to the control group. When co-transfecting pCI-neo-p17 with pcDNA3.1-TKS5 or pcDNA3.1-Rab40b, the p17-mediated downregulation of TKS5 and Rab40b was partially reversed (TKS5: 3.1± 0.15-fold; Rab40b: 2.8± 0.12-fold compared to p17 alone; p< 0.01, n = 3), and this reversal also moderately restored MMP9 expression (3.2± 0.13-fold; p <0.01; **Figures 5B, C**). However, the p17-induced decrease in Nck1 levels remained unaffected by co-transfection (1.01± 0.09-fold, p= 0.34), indicating that Nck1 is independently regulated by p17. These findings confirm that p17 inhibits MMP9 expression by suppressing TKS5 and Rab40b, which impairs invadopodia formation and reduces cell migratory potential. Interestingly, in cells treated with an Akt inhibitor (Akt inhibitor III), expression levels of TKS5 and MMP9 were significantly reduced (TKS5: 0.10± 0.08-fold; MMP9: 0.10± 0.06-fold compared to untreated controls; p<0.01; **Figure 5D**), suggesting that the PI3K-Akt pathway positively regulates TKS5 and MMP9 expression. “This finding is consistent with a previous study indicating that PTEN can dephosphorylate PIP3 and FAK, thereby inhibiting cell growth, invasion, migration, and the formation of focal adhesions (Tamura et al., 1999a).” Immunoblots from **Figures 5A–D** were quantitated by densitometric analysis using ImageJ software (**Supplementary Figure S2**).

### 3.7 The p17 transcriptionally downregulates TKs5, Nck1, and Rab40b genes and reduces the complex formation of TKs5-Nck1 and TKs5-Rab40b

**Figure 6:**
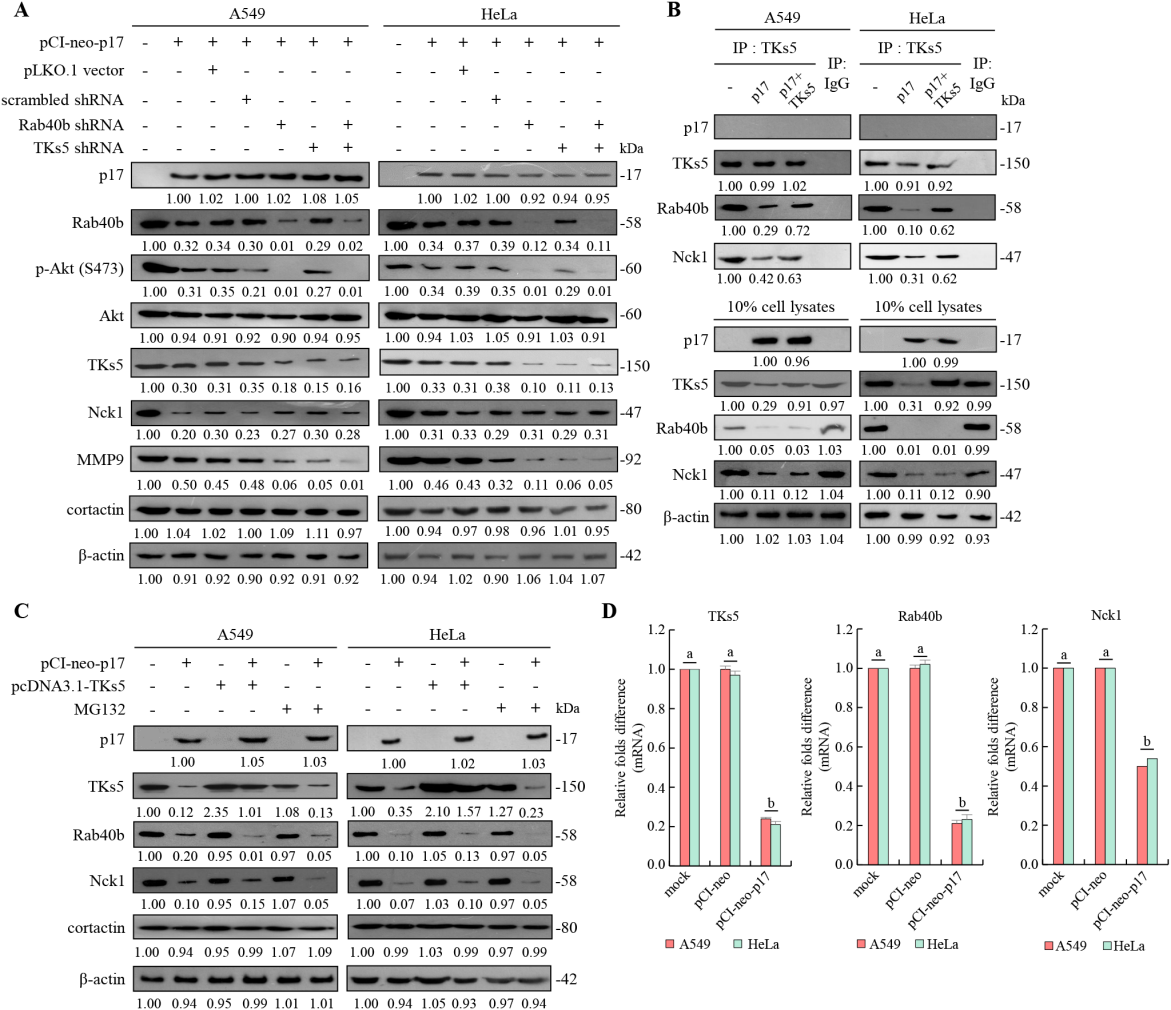
The p17 protein of ARV downregulates TKs5, Rab40b, and Nck1 genes and reduces the formation of TKs5-Rab40b and TKs5-Nck1 complexes. **(A)** The expression levels of TKs5, Rab40b, Nck1, MMP9, and cortactin in pcDNA3.1-transfected and p17-transfected cancer cells were examined. Whole-cell lysates were collected at 24 hr post-transfection for Western blot assays. β-actin was included as a loading control. The fold activation and inactivation indicated below each lane were normalized against those of mock transfection, which were considered 1-fold. **(B)** To examine whether p17 modulates the formation of the TKs5-Nck1 TKs5-Rab40b complexes, a co-immunoprecipitation assay was performed in cells that were either transfected with p17 or co-transfected with p17 and TKs5. **(C)** The p17 plasmid DNA was transfected into different cancer cells, and the proteasome inhibitor MG132 was used to inhibit protein degradation. Cell lysates were collected after 24 hours, and the Western blot was used to analyze the expression levels of TKs5, Rab40b, and Nck1. Immunoblots from **Figures 5A–D** were quantitated by densitometric analysis using ImageJ software. Data in panels **(A-D)** are means and SE from three independent experiments **Supplementary Figure S3**). **(D)** The mRNA levels of TKs5, Rab40b, and NcK1 genes in mock, vector, and p17-transfected cells were analyzed by qRT-PCR. The levels of the mock group in cells alone were considered to be 1-fold. Each value represents mean± SE from three independent experiments, determined using Duncan’s multiple range test. Similar letters (a, b, c) denote no significance at p < 0.05. Immunoblots from panels.

To further investigate whether the depletion of TKs5 and Rab40b affects downstream MMP9, shRNAs targeting TKs5 and Rab40b were utilized. The results demonstrated that p17 significantly reduced MMP9 protein levels (0.42± 0.05-fold vs. control; p<0.01, n= 3). This inhibitory effect was further enhanced in TKS5 and Rab40b knockdown cells, where MMP9 expression decreased to 0.05± 0.04-fold and 0.05± 0.03-fold, respectively, compared to control (p<0.01; **Figure 6A**). A previous study suggested that PIP_(3,4)2_ may recruit the scaffolding protein TKs5 by binding to its PX domain, thereby facilitating its localization to the cell membrane in conjunction with cortactin (Clark and Weaver, 2008). Once localized, TKs5 is believed to regulate invadopodia formation by utilizing its third SH3 domain to bind key actin regulators, including Nck1, Nck2, N-WASP, and Grb2 (Yamaguchi et al., 2005). To explore whether the p17 protein regulates the formation of the TKs5-Nck1 and TKs5-Rab40b complexes, co-immunoprecipitation was used to determine its protein interactions. Our co-immunoprecipitation data further showed that p17 markedly reduced the levels of TKS5-Nck1 and TKS5-Rab40b complexes (TKS5-Nck1: 0.35± 0.06-fold; TKS5-Rab40b: 0.18± 0.05-fold vs. control; p< 0.01). These inhibitory effects were moderately reversed in cells co-transfected with pCI-neo-p17 and pcDNA3.1-TKS5, with complex levels restored to approximately 0.65± 0.07-fold (p<0.05 vs. p17 alone; **Figure 6B**). The results indicate that p17 inhibits the formation of the TKs5-Nck1 and TKs5-Rab40b complexes.

After demonstrating that the p17 protein downregulates TKs5, Rab40b, and Nck1, we further investigated whether the reduction in TKs5, Nck1, and Rab40b levels regulated by p17 was mediated by the ubiquitin-proteasome degradation pathway. To this end, we employed the protease inhibitor MG132 to assess its effect on the expression levels of these proteins. To assess whether protein degradation contributed to this effect, we treated cells with MG132, a proteasome inhibitor. No significant restoration of TKS5, Rab40b, or MMP9 protein levels was observed in MG132-treated, p17-transfected cells compared to the p17-only group (p>0.05; **Figure 6C**), indicating that p17 does not promote proteasomal degradation of these proteins. Immunoblots from **Figures 6A–C** were quantitated by densitometric analysis using ImageJ software (**Supplementary Figure S3**). To investigate further whether p17 transcriptionally downregulates the TKs5, Nck1, and Rab40b genes, the mRNA levels of these genes in p17-transfected cancer cells were quantified using qRT-PCR. qRT-PCR analysis revealed that mRNA levels of TKS5, Rab40b, and MMP9 were significantly decreased in p17-transfected cells (TKS5: 0.25± 0.06-fold, Rab40b: 0.22± 0.05-fold, Nck1: 0.50± 0.04-fold; p<0.01; **Figure 6D**), suggesting that p17 acts at the transcriptional level to downregulate these genes.

### 3.8 The p17 protein inhibits the formation of invadopodia in A549 and HeLa cancer cells

**Figure 7:**
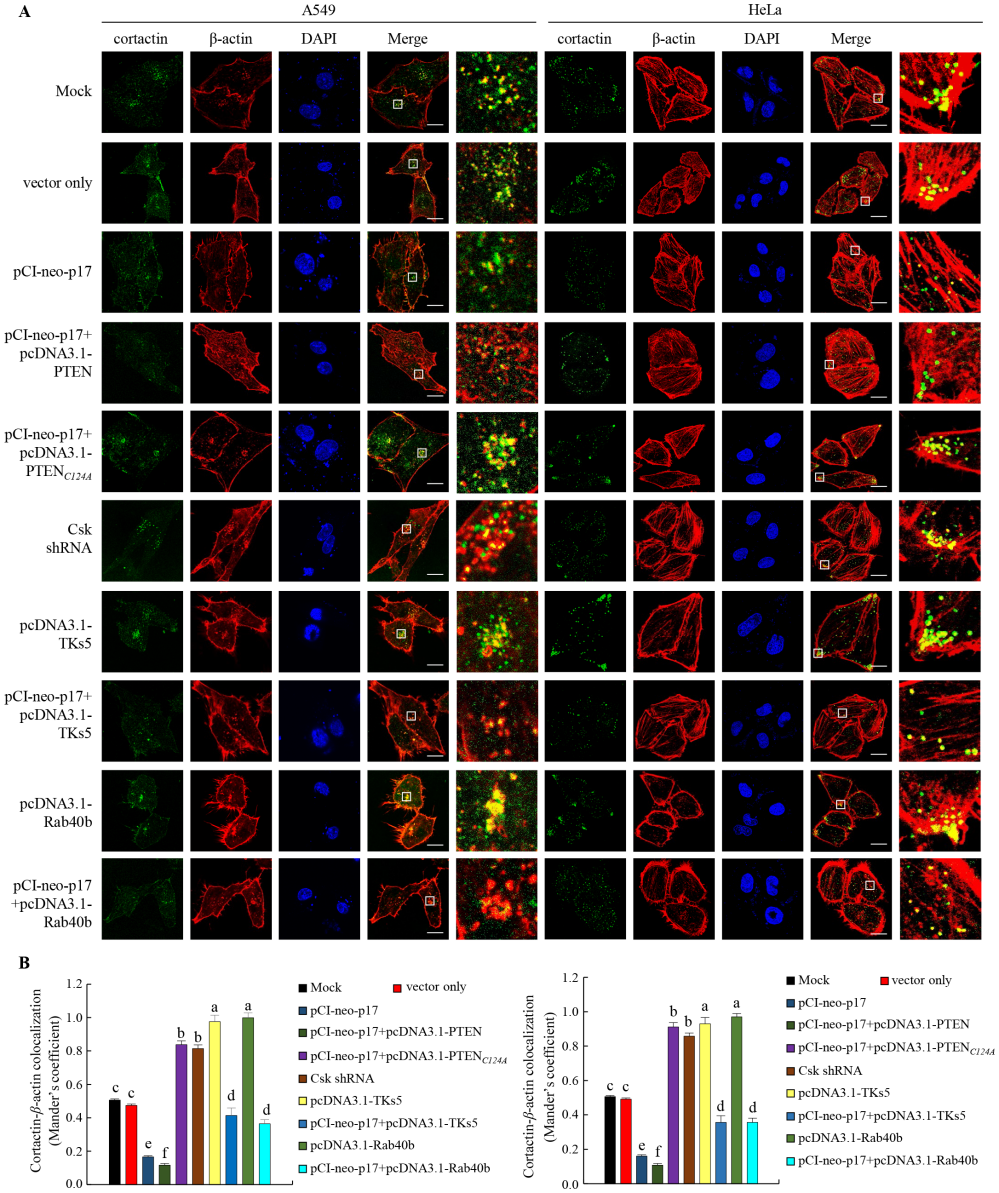
The p17 protein of ARV inhibits invadopodia formation in A549 and HeLa cancer cells, impairing their migration ability. **(A, B)** The role of PTEN or the PTEN (C124A) mutant protein in p17-transfected A549 and HeLa cells was investigated. After transfection, the cells were fixed and subjected to immunofluorescence staining with DAPI. The cytoskeletal proteins ß-actin (red) and cortactin (green) were visualized using a fluorescence microscope. Invadopodia, which are structures composed of cortactin and ß-actin, were identified by the appearance of yellow dots in the merged images, indicating their localization beneath the cells. Colocalization of ß-actin and cortactin in panel A was measured **(B)**.

**Figure 8:**
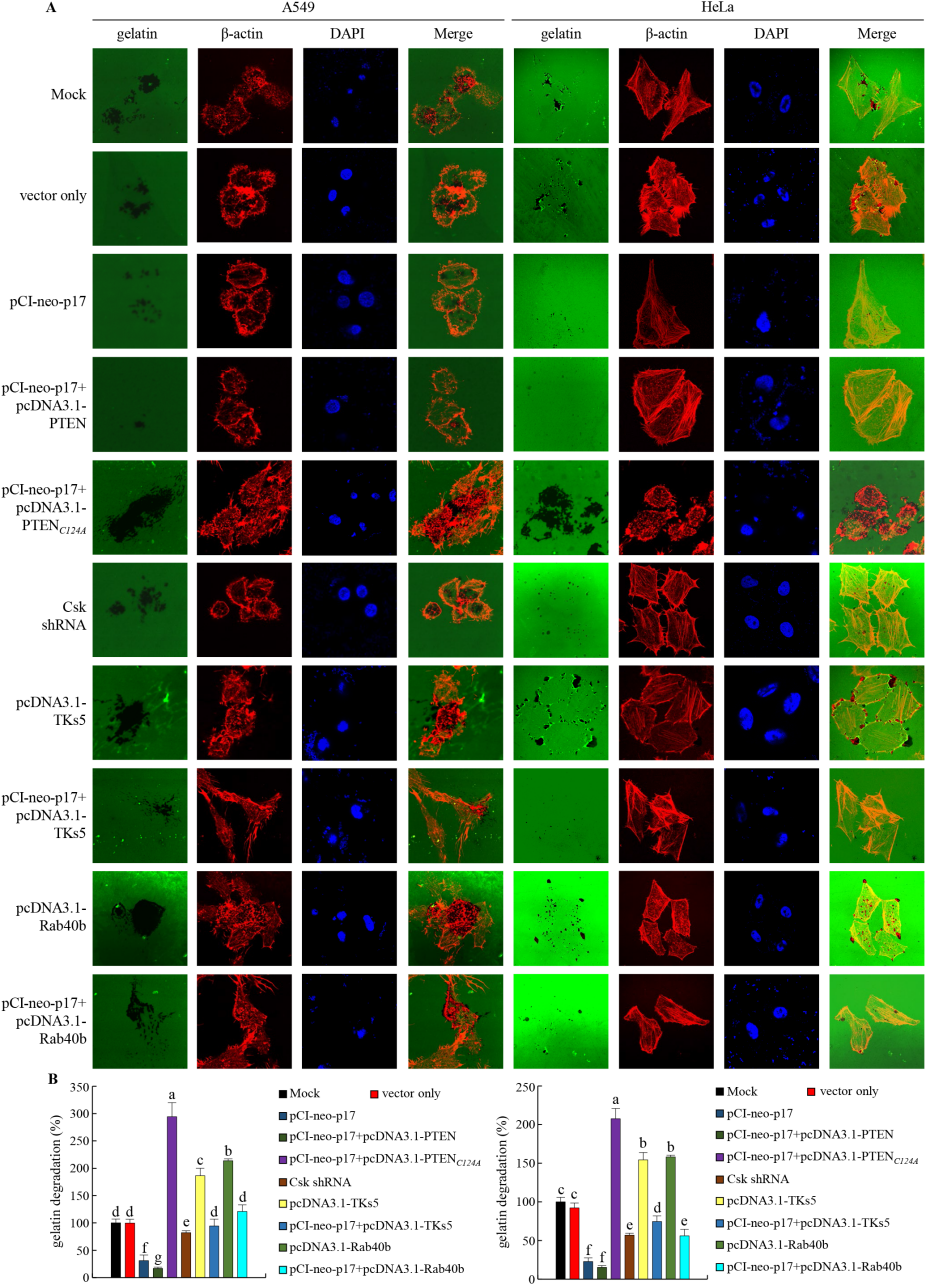
The p17 protein of ARV inhibits the ability of invadopodia in A549 and HeLa cells to degrade the extracellular matrix (ECM). **(A, B)** The effects of PTEN or the PTEN (C124A) mutant protein were examined in A549 and HeLa cancer cells transfected with p17. After transfection, the cells were fixed and subjected to immunofluorescence staining with DAPI. ß-actin (red) and gelatin (green) were visualized using a fluorescence microscope. Areas where the ECM had been degraded appeared as black regions, indicating the presence of functional invadopodia. The areas where the matrix had been degraded, leaving behind regions without fluorescence, were recorded and measured **(B)**.

Having demonstrated that p17 transcriptionally downregulates TKs5, Nck1, and Rab40b and reduces the formation of TKs5-Nck1 and TKs5-Rab40b complexes, we next examined whether p17 can inhibit the formation of invadopodia. To investigate this, immunofluorescence staining combined with gelatin degradation assays were utilized to conduct colocalization analysis of cortactin and *β*-actin. This experimental design will help assess whether p17 significantly affects the interaction between cortactin and actin, thereby inhibiting the assembly and formation of invadopodia and providing deeper insights into its potential inhibitory role in cell migration and invasion. Our results demonstrated that transfection with pCI-neo-p17 led to a significant reduction in invadopodia formation, with the number of invadopodia per cell decreasing by approximately 65% compared to the control group (p <0.01, n= 3; **Figures 7A, B**). Co-transfection with pCI-neo-p17 and pcDNA3.1-PTEN further suppressed invadopodia formation, reducing the number by 80% compared to control (p<0.001), suggesting an additive inhibitory effect through PTEN overexpression. In contrast, co-expression of p17 with PTEN-C124A, a lipid phosphatase-inactive mutant, partially rescued invadopodia formation to about 70% of control levels (p<0.05 vs. p17+PTEN; p>0.05 vs. control), indicating the critical role of PTEN’s phosphatase activity. Similarly, co-expression with TKS5 significantly restored invadopodia number (~75% of control, p<0.05 vs. p17 alone). In the gelatin degradation assay, which measures extracellular matrix (ECM) degradation mediated by invadopodia, cells transfected with pCI-neo-p17 showed a marked reduction in matrix degradation, with degradation areas decreasing to ~20% of the control group (p<0.01, n =3; **Figures 8A, B**). In contrast, cells co-transfected with p17 and PTEN-C124A, TKS5, or Rab40b exhibited significantly increased matrix degradation, with dark zones indicating active ECM breakdown restored to 65-80% of control levels (p <0.05 vs. p17-only group; **Figures 8A, B**). These dark areas represent regions where the fluorescently labeled gelatin substrate was digested by functional invadopodia. These findings support the conclusion that ARV p17 inhibits both the formation and function of invadopodia. Moreover, the partial rescue by PTEN-C124A, TKS5, or Rab40b suggests that PTEN’s lipid phosphatase activity, along with TKS5 and Rab40b signaling, are crucial for invadopodia-mediated ECM degradation. In cells co-expressing the p17 protein, invadopodia formation was reversed. Notably, knockdown of Csk rescued the p17-mediated suppression of invadopodia formation, as evidenced by restored TKs5 puncta formation and increased degradation of the fluorescent gelatin matrix. In summary, our results indicate that the p17 protein inhibits invadopodia formation by activating the p53-PTEN pathway to suppress the FAK-Src pathway and by downregulating TKs5, Rab40b, Nck1, and MMP9.

## 4 Discussion

Previous studies have highlighted that the ARV p17 protein is a potent suppressor of nucleoporin Tpr, resulting in the nuclear accumulation of p53 in Vero cells (Huang et al., 2015). This accumulation triggers the activation of downstream effectors, including p21 and PTEN, to promote autophagy, cell cycle arrest, and tumor suppression (Chi et al., 2013; Chiu et al., 2016, 2018; Li et al., 2022; Hsu et al., 2024). In this study, we expand upon these findings by demonstrating that the same Tpr-p53-PTEN signaling axis is also activated by p17 in HeLa and A549 cancer cells. This highlights the potential tumor-suppressive role of p17 in malignancies with diverse genetic backgrounds. Beyond these effects, p17 has demonstrated its ability to modulate CDK-cyclin complexes, leading to arrest cell cycle and inhibiting the proliferation of cancer cells (Chiu et al., 2016). Additionally, p17 has been observed to suppress angiogenesis through the promotion of dipeptidyl peptidase 4 (DPP4) secretion, an enzyme critical to blood vessel formation and maintenance (Manocha et al., 2021). By exerting these multifaceted regulatory effects on cancer cell behavior, cell cycle control, tumor growth inhibition, reduced angiogenesis, and suppression of invasion pathways, p17 emerges as a potential candidate for anticancer therapies. These findings open new avenues for leveraging p17 in developing therapeutic strategies to target and disrupt cancer progression at multiple levels.

This study suggests that ARV p17 protein functions as a negative regulator of TKs5-Nck1 and TKs5-Rab40b complexes, which impacts invadopodia formation in HeLa and A549 cancer cells (Jacob et al., 2016). Our findings reveal that the p17 protein suppresses invadopodia formation in A549 and HeLa cancer cells through several pathways. Notably, p17 activates the p53-PTEN signaling pathway, resulting in the dephosphorylation of FAK at the Y397 site. This dephosphorylation hinders Src from associating with FAK at Y397, which in turn prevents Src from phosphorylating TKs5. Phosphorylation of TKs5 at this site is a key requirement for facilitating cell migration and the formation of invadopodia (Tamura et al., 1998; Saykali and El-Sibai, 2014). p17 modulates MMP9 activity, underscoring its role in curbing the invasive behavior of cancer cells (Jacob and Prekeris, 2015). TKs5 plays a role in both the expression and activity of MMP9, indicating that TKs5 is essential for MMP9 secretion. Loss of TKs5 results in lower MMP9 levels and reduced invasive capacity in cancer cells (Stylli et al., 2014; Jacob et al., 2016). FAK signaling is crucial for processes such as matrix adhesion and cell migration (Mitra et al., 2005; Chan et al., 2009; Zhao and Guan, 2011). PTEN modulates cell adhesion and migration by regulating FAK activity. FAK is vital for the formation of focal adhesions, where integrins interact with the ECM. When PTEN is active, it dephosphorylates FAK, disrupting the signaling needed to sustain focal adhesions and thus diminishing the migratory and invasive potential of cancer cells (Tamura et al., 1998, 1999; Schwab and Stock, 2014). Notably, FAK is frequently overexpressed in various cancers, with high FAK expression levels associated with poor prognosis and increased metastasis (Chuang et al., 2022; Hu et al., 2024). Clinical studies provide substantial evidence that FAK mRNA transcript levels are significantly elevated in multiple malignant tumors, showing a 25% to 37% increase compared to normal tissues (Sulzmaier et al., 2014). Given FAK’s pivotal role in cancer progression, interest in targeting FAK as a therapeutic strategy has increased. Consequently, recent years have seen significant efforts to develop anticancer drugs that inhibit FAK activity, disrupt related pathways, and reduce tumor growth and metastasis (Dunn et al., 2010). TKs5 also plays a crucial role in the formation of invasive pseudopodia, which are essential for cancer cell invasion and metastasis. The TKs5 protein regulates the production of ROS mediated by NADPH oxidase, thereby promoting the formation of pseudopodia or invasive podium-like structures, which are critical for cancer cells to penetrate tissues and enable metastasis (Diaz et al., 2009). An earlier study has shown that TKs5 and FGD1 form a complex that plays a crucial role in cell migration by facilitating the activation of CDC42. Activated CDC42, in turn, influences cytoskeletal dynamics and promotes efficient cell movement (Huang et al., 2015). When TKs5, FGD1, or CDC42 functions are disrupted, the proper activity of membrane-type 1 matrix metalloproteinase (MT1-MMP) is compromised. This disruption prevents the formation of collagenolytic pseudopodia and impedes ECM degradation. Consequently, tumor cells’ directional migration and invasion speed through the ECM are significantly reduced (Zagryazhskaya-Masson et al., 2020). These findings suggest that the TKs5-FGD1 complex and CDC42 activation are essential for MT1-MMP-mediated matrix remodeling and cell invasion, highlighting their potential as therapeutic targets to limit tumor cell invasiveness and metastatic potential.

Specifically, we found that p17 transcriptionally downregulates *TKs5*, *Nck1*, and *Rab40b*, leading to reduced formation of the TKs5-Nck1 and TKs5 Rab complexes and consequently inhibiting invadopodia formation. While our findings demonstrate this regulatory effect, the precise mechanisms by which it modulates the transcriptional repression of these genes remain unclear. We acknowledge that this aspect is currently speculative. Future studies will aim to identify the specific transcription factors, epigenetic regulators, and chromatin modifiers involved in this process. Based on the known role of p17 in activating the p53 pathway, we propose that transcriptional repressors or chromatin modifiers such as p53 itself, ZEB1/2 (Wels et al., 2011), Snail, Slugor the histone-modifying enzymes HDAC and EZH2 may be involved (Li et al., 2021). Other candidates like the transcriptional repressors REST and YY1, which can interact with p53 or polycomb repressive complexes, also warrant investigation. A previous report has suggested that TKs5 interacts with Rab40b or Nck1 to regulate the initiation and maturation of invadopodia, structures crucial for cancer cell invasion and metastasis (Hoshino et al., 2012). In rat breast tumor cells, Nck1 activity underscores its essential role in cell membrane protrusion and invasive behavior (Yamaguchi et al., 2005; Chaki et al., 2013). TKs5 facilitates invadopodia formation by binding to critical actin regulators like Nck1. Furthermore, beyond the p53-PTEN and FAK-Src-TKs5 signaling pathways, our findings reveal a novel mechanism through which p17 inhibits the PI3K/Akt pathway (Eddy et al., 2017). PI3K phosphorylates PIP(3,4)_2_ to produce PIP3, which subsequently recruits kinases such as Akt, crucial for regulating invadopodia formation (Cantley, 2002). A recent study demonstrated that Rab40b can specifically recognize and bind to TKs5 at the tips of invadopodia, playing a critical role in the delivery of MMP-containing vesicles that facilitate invadopodia expansion. Through this interaction, TKs5 enables Rab40b to mediate the trafficking and localization of MMP2 and MMP9 to invadopodia, which is essential for their formation and function (Jacob et al., 2016). This coordinated transport of MMPs supports the invasive potential of cancer cells by ensuring that invadopodia are equipped with the necessary enzymes for matrix degradation and cellular invasion.

Previous studies have demonstrated that highly invasive cancer cells develop protrusive structures known as invadopodia, which are rich in actin filaments and play a significant role in degrading the extracellular matrix (Gould and Courtneidge, 2014). Invadopodia are mainly comprised of *β*-actin and cortactin, which are surrounded by a ring of actin-binding proteins such as N-WASP and cofilin (Yamaguchi et al., 2005). These structures penetrate the ECM effectively, enabling its breakdown and facilitating cell migration. In this study, invadopodia were identified through immunofluorescence staining of *β*-actin and cortactin, and gelatin degradation assays were performed to assess their activity. Our findings indicate that the ARV p17 protein restricts both cell migration and invadopodia formation. It has been suggested that once cofilin and Arp2/3 complexes polymerize with downstream proteins and actin, cortactin undergoes dephosphorylation, a step critical for stabilizing the invadopodia precursor (Yamaguchi et al., 2005). This stabilization, in turn, is crucial for the formation of invadopodia (Oser et al., 2009). The study suggests several avenues for future research to deepen our understanding of p17’s mechanisms and therapeutic potential. One promising direction would be to investigate the interaction of p17 with cofilin and Arp2/3 complexes, as these complexes play a critical role in the dephosphorylation of cortactin, which is essential for stabilizing invadopodium precursors and subsequent invadopodia formation (Yamaguchi et al., 2005). Understanding how p17 may influence these interactions could shed light on its precise role in inhibiting invadopodia formation. Additionally, exploring the potential interplay between p17 and other cellular pathways involved in cancer progression, such as those related to cell cycle regulation, angiogenesis, and immune responses, reveals additional therapeutic targets and strategies.

## 5 Conclusions

**Figure 9:**
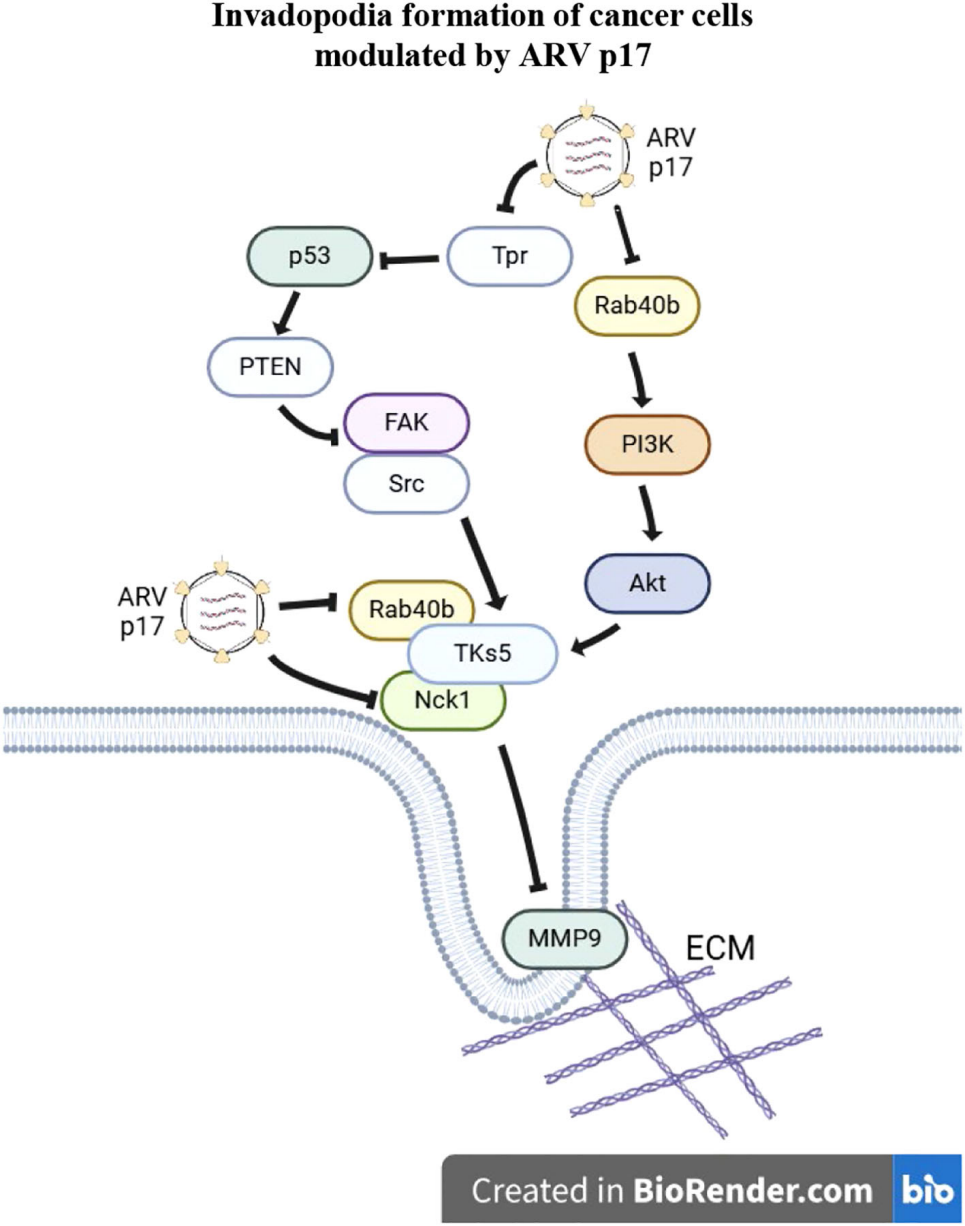
A model illustrating how the ARV p17 protein inhibits invadopodia formation in cancer cells. The ARV p17 protein suppresses nucleoporin Tpr, leading to activation of the p53-PTEN pathway. This cascade results in the downregulation of both the FAK-Src and Rab40b-PI3K-Akt signaling pathways, thereby inhibiting the formation of the TKS5-Nck1 and TKS5-Rab40b complexes. This suppression disrupts the assembly of invadopodia and associated structures required for cancer cell migration and invasion. In the diagram, arrows (→) indicate activation, while perpendicular lines (⊥) represent inhibition. To distinguish the regulatory mechanisms, dashed arrows represent transcriptional effects and solid arrows indicate post-translational regulatory events. Together, this model highlights the multifaceted role of p17 in repressing cancer cell invasiveness through both gene expression and protein-level control.

In conclusion, our study provides new insights into the role of the ARV p17 protein in regulating cancer cell migration. We demonstrate that p17 inhibits cell migration by modulating pseudopod formation, a critical step in cancer cell invasion. This regulation occurs through the activation of the p53-PTEN pathway and the inactivation of the Rab40b-PI3K-Akt pathway, which leads to the suppression of the formation of TKS5-Nck1 and TKS5-Rab40b complexes. These findings suggest that p17 impedes invadopodia formation, a key factor in tumor metastasis, by disrupting the molecular interactions that drive cell migration and invasion. Our results offer a clearer understanding of the molecular mechanisms underlying virus-induced changes in cancer cells, shedding light on how viruses, such as ARV, manipulate host cellular pathways to modulate cell behavior. A proposed model illustrating how p17 modulates the suppression of the TKS5-Rab40b and Nck1-TKS5 complexes to impede invadopodia formation is shown in **Figure 9**.

### 5.1 Future prospects

Looking ahead, it will be important to further explore the therapeutic potential of p17 in cancer treatment. Future studies should focus on the *in vivo* validation of our findings, assessing the long-term effects of p17 on tumor growth, metastasis, and host immune responses. Additionally, investigating the interactions between p17 and other key molecular pathways involved in cancer progression could reveal new targets for therapeutic intervention. Understanding how p17 affects various cancer types and exploring its combination with other therapies may provide a promising avenue for enhancing cancer treatment strategies.

### 5.2 Limitations

While our study provides valuable insights, certain limitations should be acknowledged. The *in vitro* experiments require further validation in animal models to confirm the clinical relevance of our findings. Additionally, the role of p17 in different cancer cell types and its potential off-target effects need to be carefully evaluated in future studies.

## Data Availability

The original contributions presented in the study are included in the article/**Supplementary Material**. Further inquiries can be directed to the corresponding author.
