# Supplementary material for: The oncolytic avian reovirus p17 protein suppresses invadopodia formation via disruption of TKs5 complexes and oncogenic signaling pathways
Source: Front Cell Infect Microbiol. 2025 Jun 12;15:1603124. doi: 10.3389/fcimb.2025.1603124 (PMC12198159; doi:10.3389/fcimb.2025.1603124)
Supplement: Supplementary file 1 [file DataSheet1.pdf]

Supplemental information

Table S1. shRNAs used in this study

	Cat. No.	Sequence (5′-3′)
Tpr	TG308677	GGTGAAGATAGTAATGAAGGAACTGGTAG
p53	TG320558	CTCAGACTGACATTCTCCACTTCTTGTTC
PTEN	TG320498	CTTGACCAATGGCTAAGTGAAGATGACAA
Rak	TG517143	TGGTCTCAAGAGGCAGACAAGTCAGTAGT
Rock-1	TG309775	CCAGAGTCAAGAATTGAAGGTTGGCTTTC
Csk	TRCN0000000804	CGAGGAGGTGTACTTTGAGAA
Rab40b	TRCN0000047529	CGGCATTGATCGATGGATTAA
TKs5	TRCN0000135150	CATCTATGAGAATGAGGGCTT

Table S2. Primers used in this study for amplification of the respective targeted genes

Gene	Accession number	Sequence (5′-3′)*	Expected size (bp)
p17 (full)	AF330703	F: CGGAATTCATGCAATGGCTCCGCCATACGA (*Eco* RI)<br>R: GCTCTAGATCATAGATCGGCGTCAAATCGC (*Xba* I)	441
PTEN	AB009903	F: GCGAAGCTTACCATGACAGCCATCATCAAAG (*Hind* III)<br>R: CGGCTCGAGTCAGACTTTTGTAATTTGTGT (*Xho* I)	1230
PTEN mutant C124A up		F: GCGAAGCTTACCATGACAGCCATCATCAAAG (*Hind* III)<br>R: CCAGTTCGTCCCTTTCCACCTTTAGCGTGAA	390
PTEN mutant C124A down		F: AGCAATTCACGCTAAAGCTGGAAAGGGACG<br>R: CGGCTCGAGTCAGACTTTTGTAATTTGTGT (*Xho* I)	840
TKs5	NM_014631.3	F1: TAGAATTCACGATGCTCGCCTACTGCGTG (*Eco* RI)<br>R1: CAAAGCCGAATGCAGGGATGTCATACTC	1922
		F2: GAGTATGACATCCCTGCATTCGGCTTTG<br>R2: GCGTCGACCTAGTTCTTTTTCTCAAGGTAG (*Sal* I)	1483
Rab40b	NP_006813.1	F: TAAAGCTTACGATGAGCGCCCTGGGCAG (*Hind* III)<br>R: GCGGATCCTTAAGAAATTTTGCAGCTGTTTC (*Bam* HI)	837
Nck1	NM_006153	F: GCAGAGAGGATGAATTATCATT<br>R: CTGAAGTTAATGGATTATTCTG	410

Underlines in each primer indicate the restriction sites.

The restriction sites were designed in the primers for cloning.

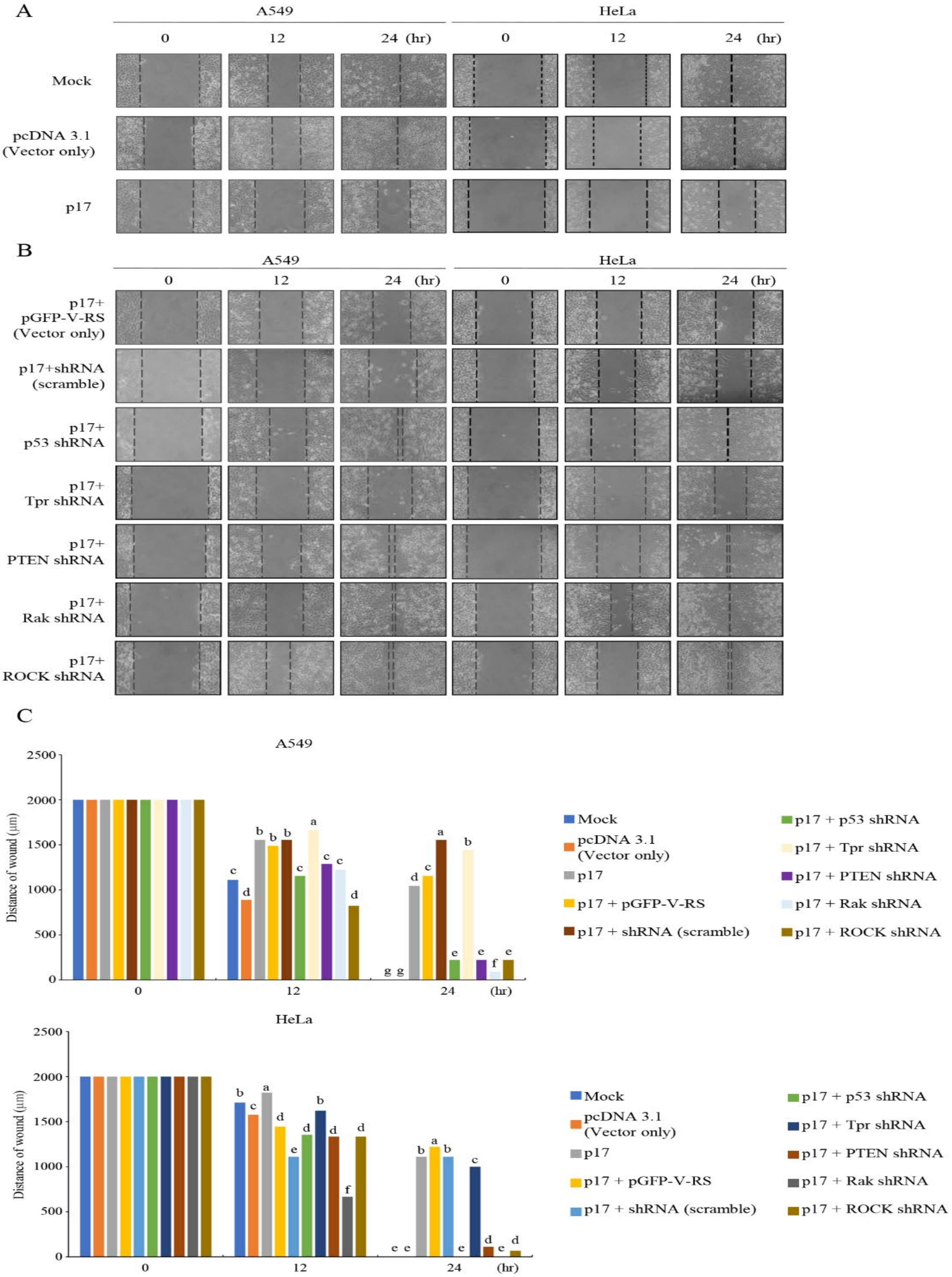
A
A549
HeLa
0
12
24 (hr)
Mock
pcDNA 3.1
(Vector only)
p17
B
p17+
pGFP-V-RS
(Vector only)
p17+shRNA
(scramble)
p17+
p53 shRNA
p17+
Tpr shRNA
p17+
PTEN shRNA
p17+
Rak shRNA
p17+
ROCK shRNA
C
Distance of wound (μm)
2500
2000
1500
1000
500
0
Mock
pcDNA 3.1
(Vector only)
p17
p17 + pGFP-V-RS
p17 + shRNA (scramble)
p17 + p53 shRNA
p17 + Tpr shRNA
p17 + PTEN shRNA
p17 + Rak shRNA
p17 + ROCK shRNA

Figure S1 The ARV p17 protein inhibits the migration of human cancer cell lines. (A)Wound-healing migration assay was performed to determine the motility of 3 transfected HeLa and A549 cancer cells. Cells were transfected with pCI-neo-p17. 4 After 12 hr of transfection, the monolayer was wounded with a sterile 200 μl pipette 5 tip and washed with culture medium. Cells were photographed by light microscope at 6 the indicated time points. It was confirmed that the p17 protein can inhibit the cell 7 migration ability of HeLa and A549 cancer cells. (B) Wound-healing migration assay 8 was performed to determine the motility of transfected HeLa and A549 cancer cells. 9 Cells were transfected with pCI-neo-p17 with or without co-transfection with p53 10 shRNA, Tpr shRNA, PTEN shRNA, Rak shRNA, and Rock shRNA. After 12 hr of 11 transfection, the monolayer was wounded with a sterile 200 μl pipette tip and washed 12 with culture medium. Cells were photographed by light microscope at the indicated 13 time points. (C)The scratch test was used to analyze the migration distances of HeLa 14 and A549 cancer cells at 0, 12, and 24 h post transfection. Scratch lines were recorded 15 at 0, 12, and 24 h, respectively. Each value represents mean± SE from three 16 independent experiments, determined using Duncan's multiple range test. Similar 17 letters denote no significance at $p < 0.05$.

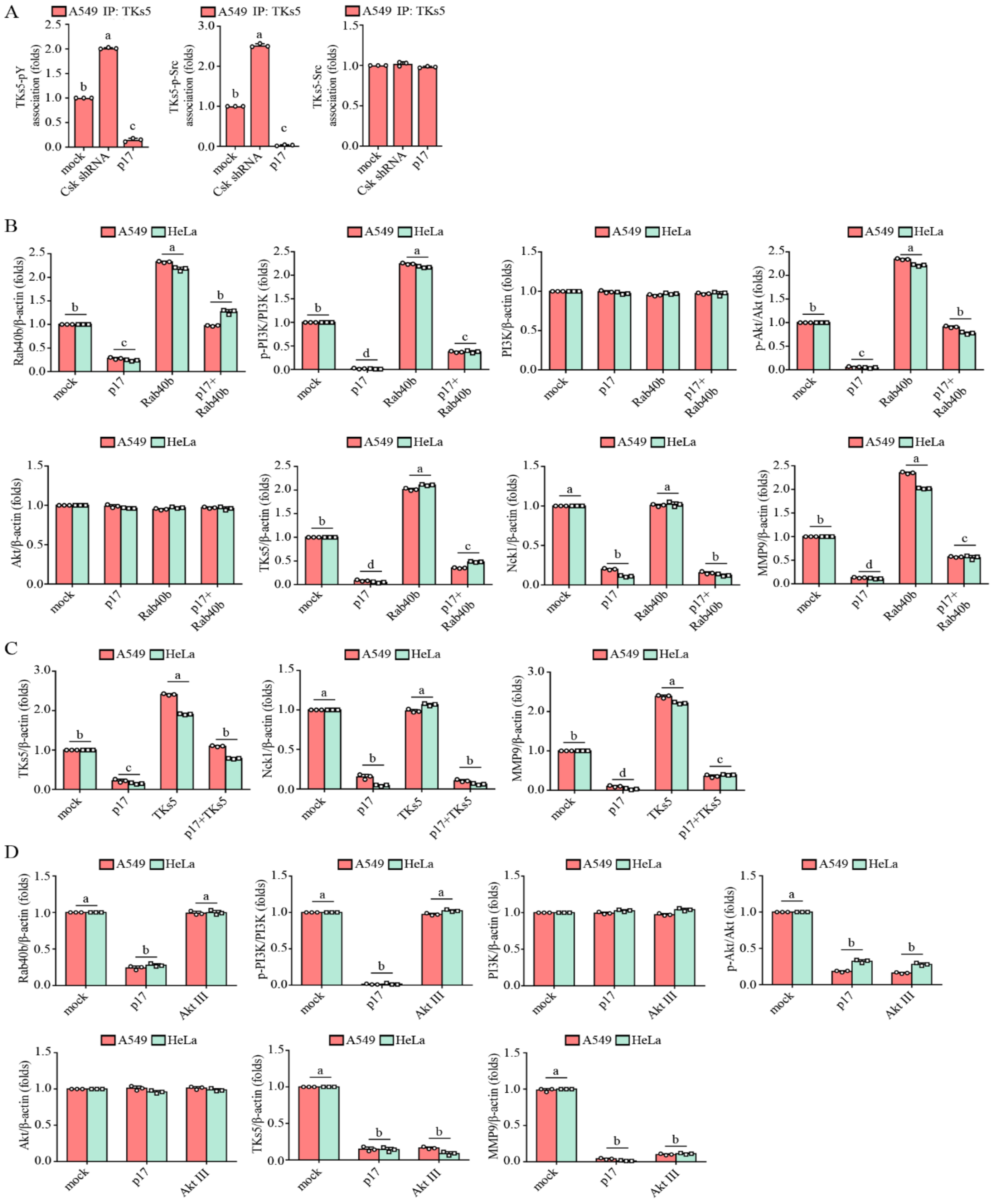

Figure S2 Immunoblots from Figure 5 (panels A-D) were quantitated by densitometric analysis using ImageJ software

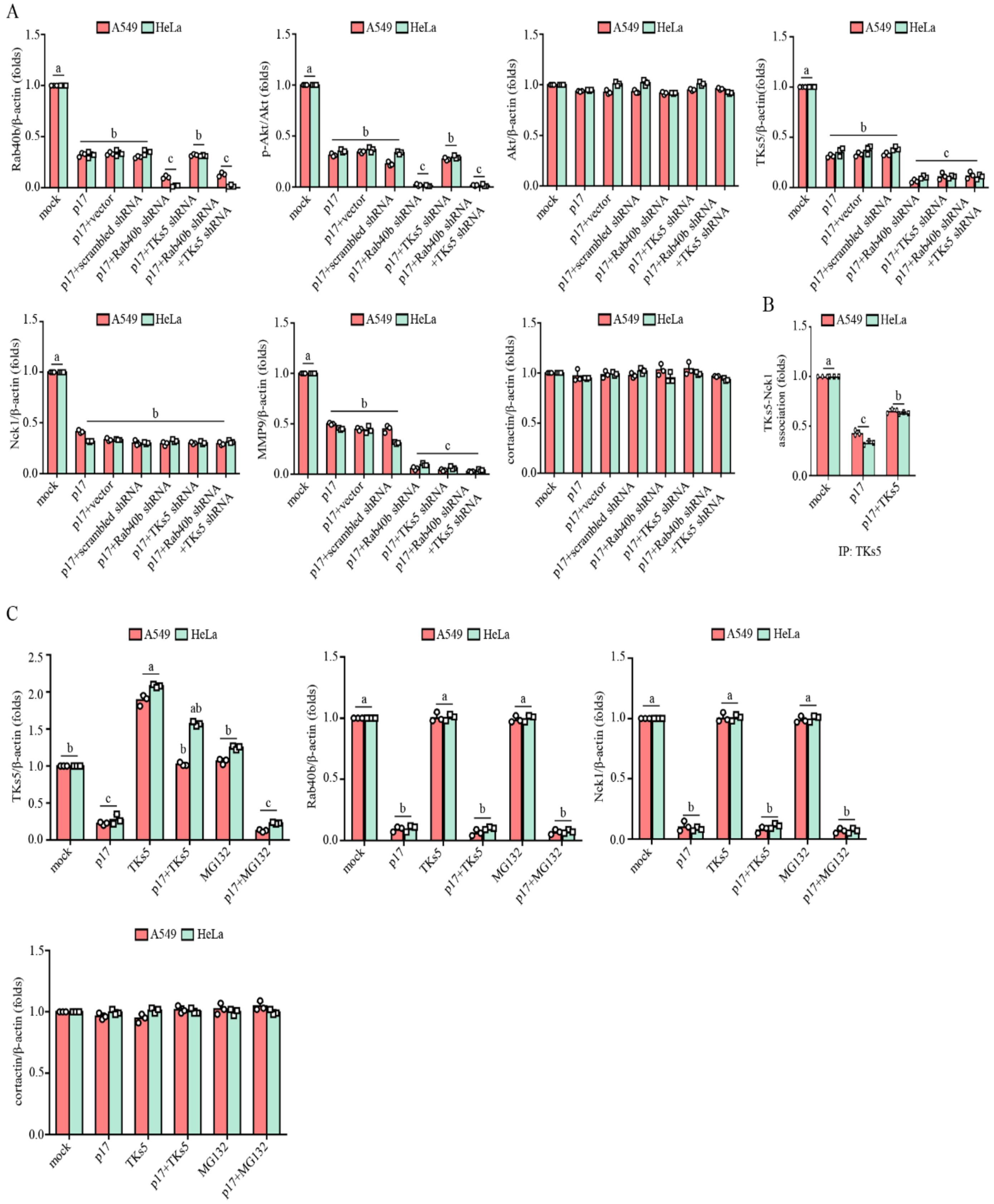

Figure S3 Immunoblots from Figure 6 panels A-C were quantitated by densitometric analysis using ImageJ software

Figure S4

Figure 1

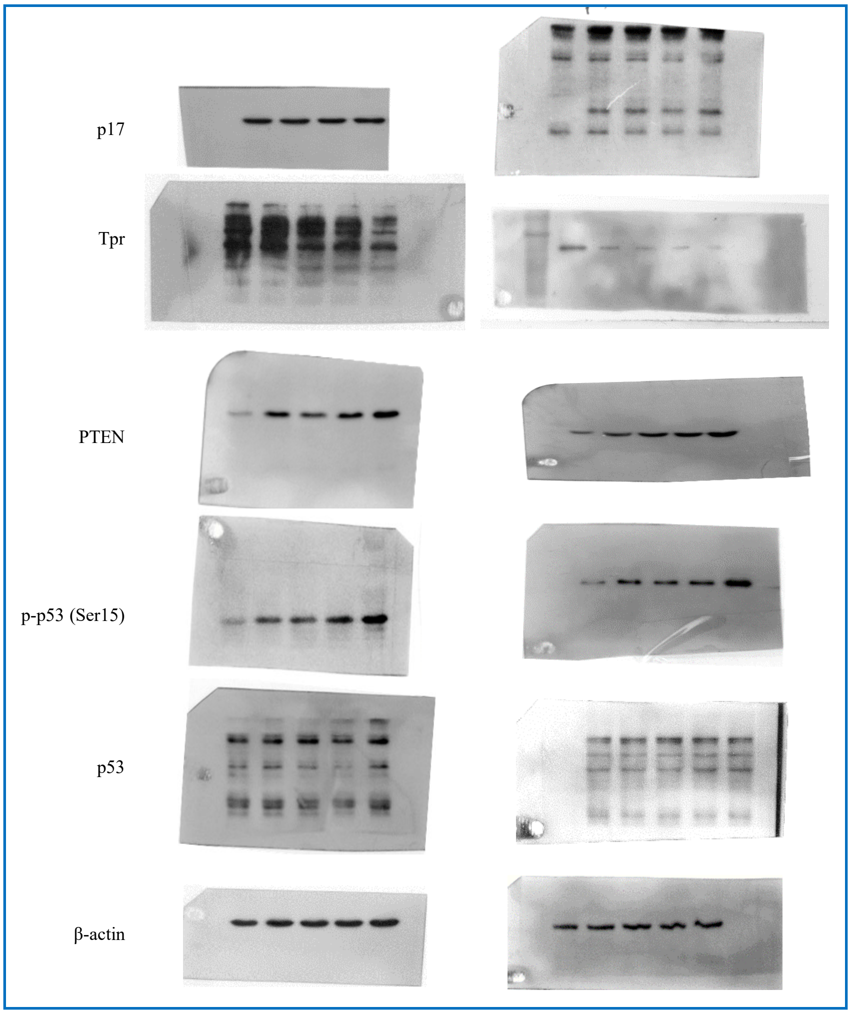

Figure 2

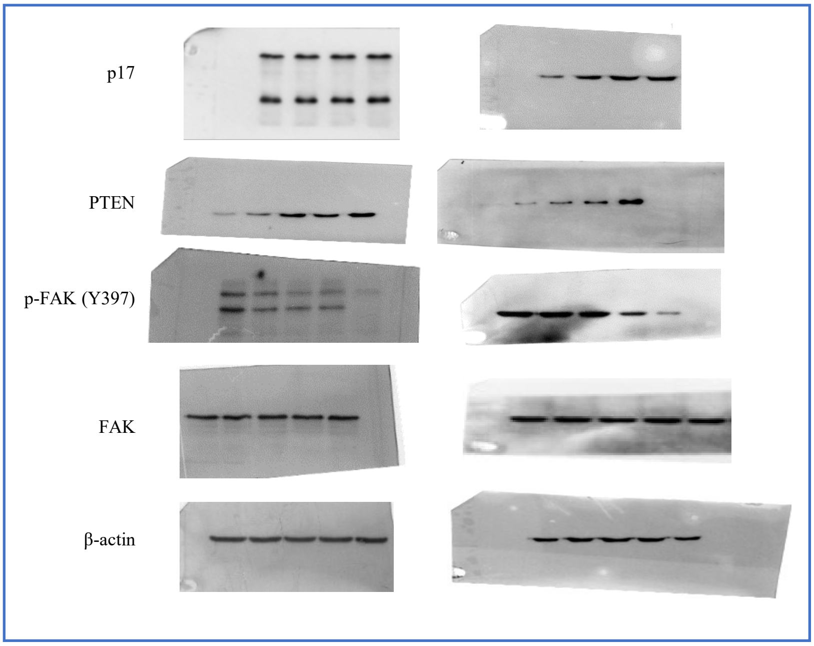

Figure 3

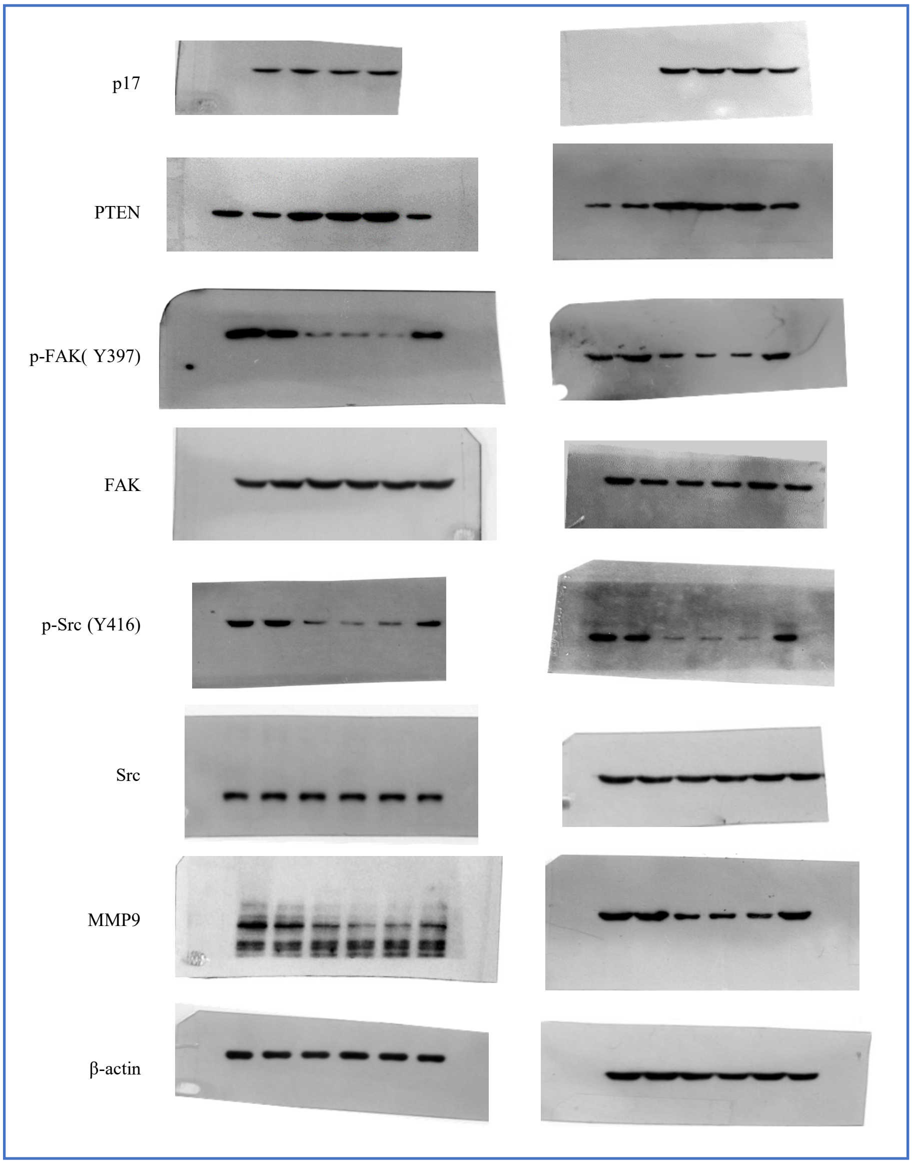

Figure 4

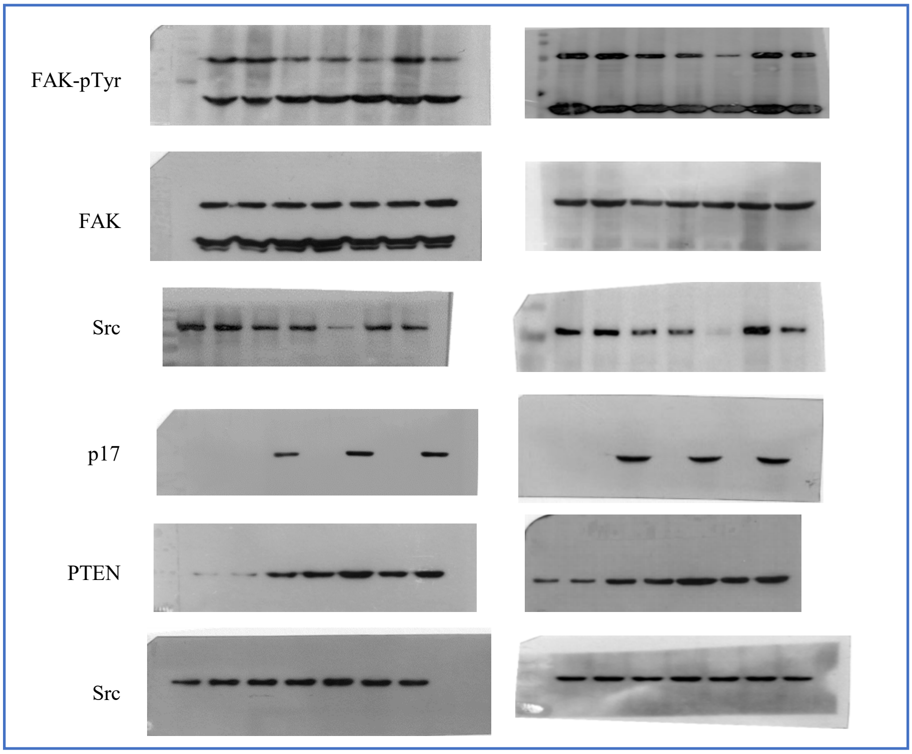

Figure 5A

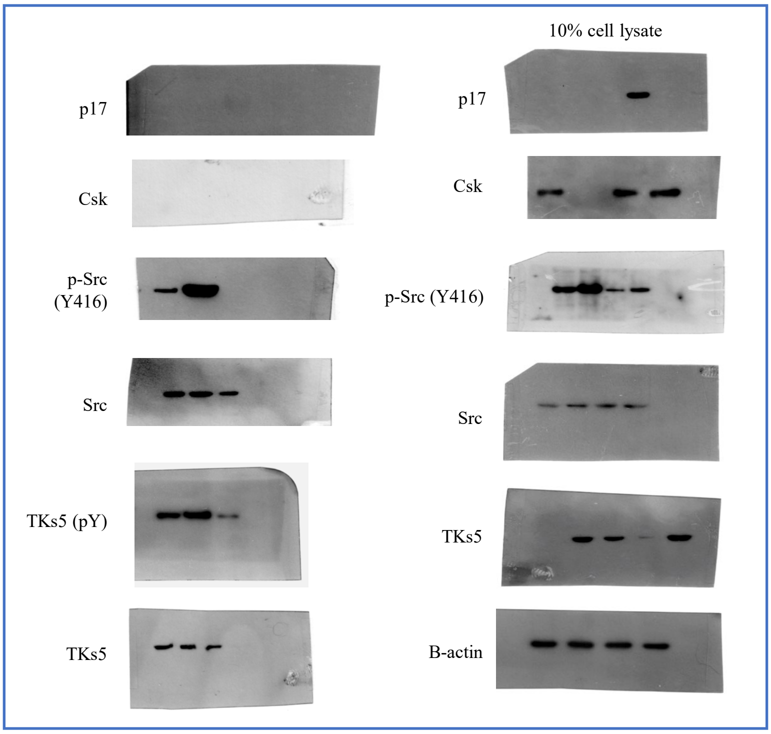

Figure 5B

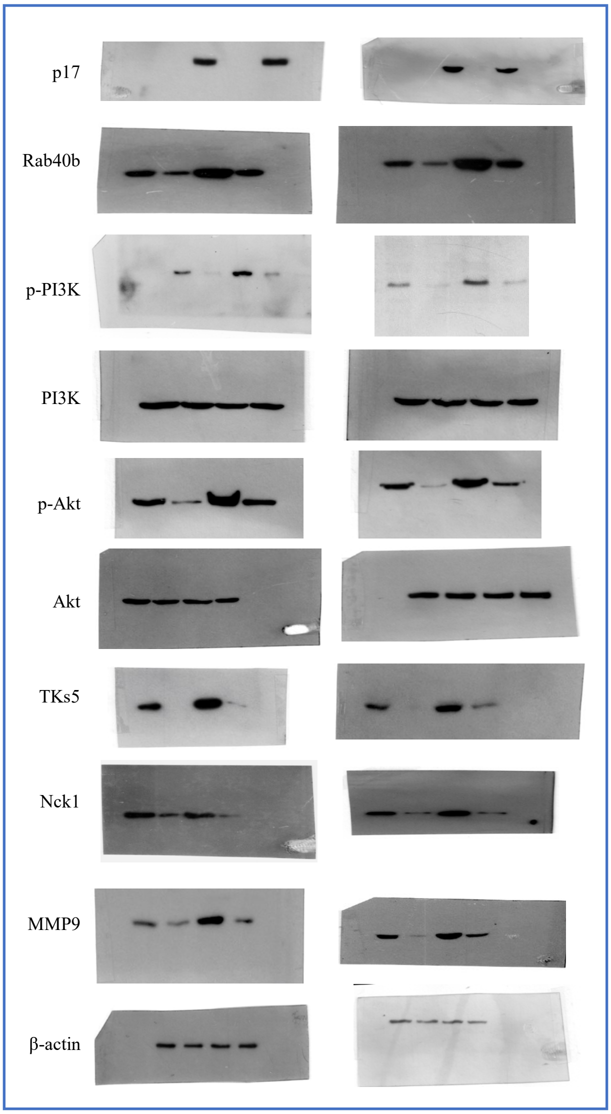

Figure 5C

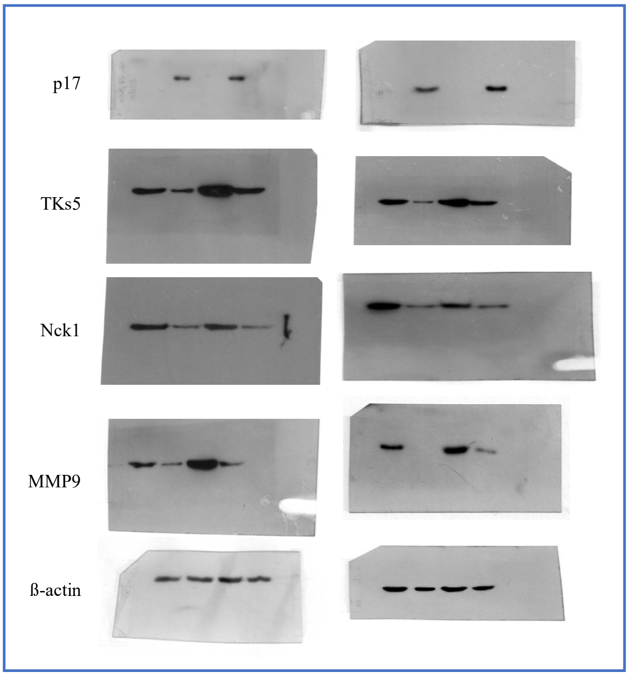

Figure 5D

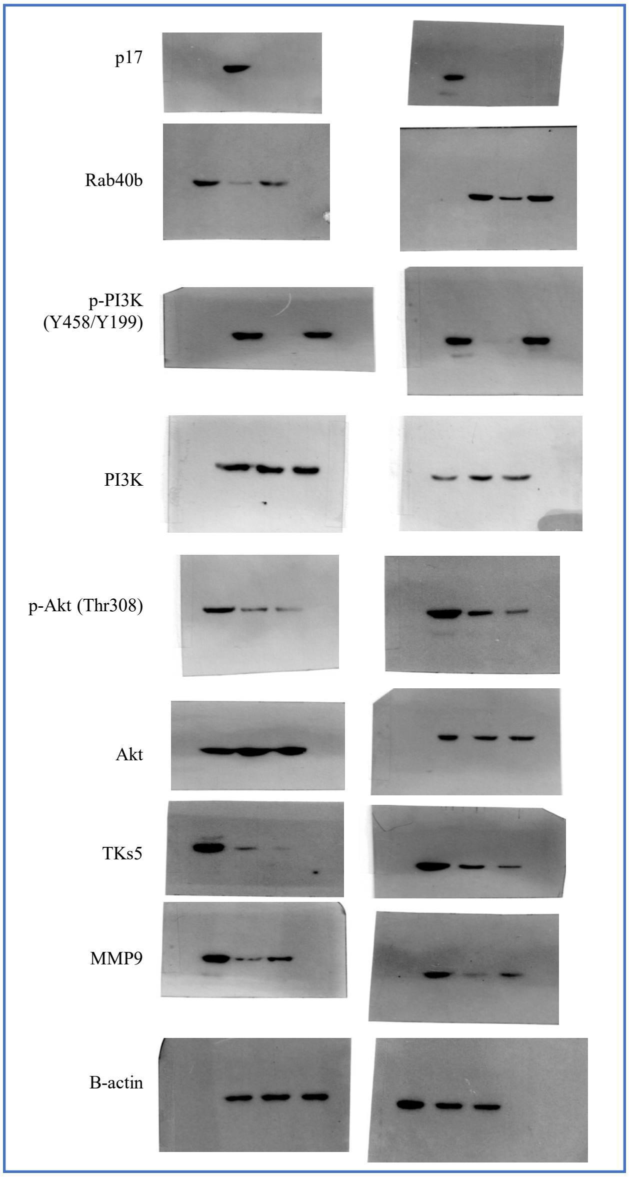

Figure 6A

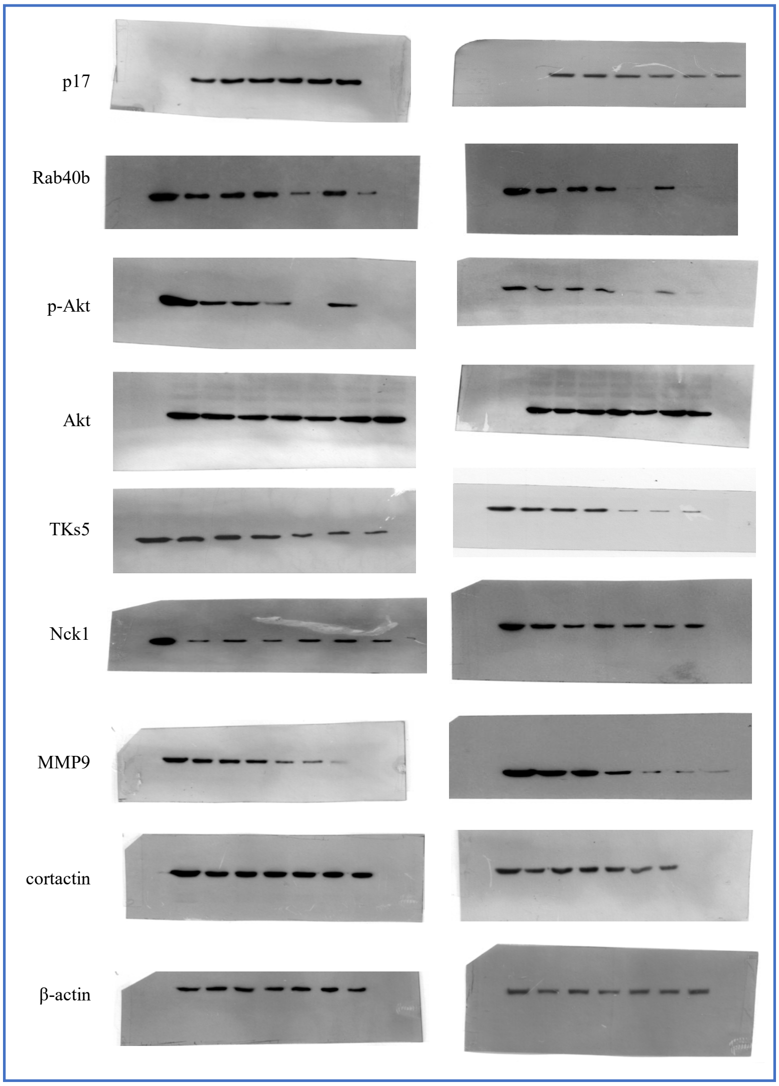

Figure 6C

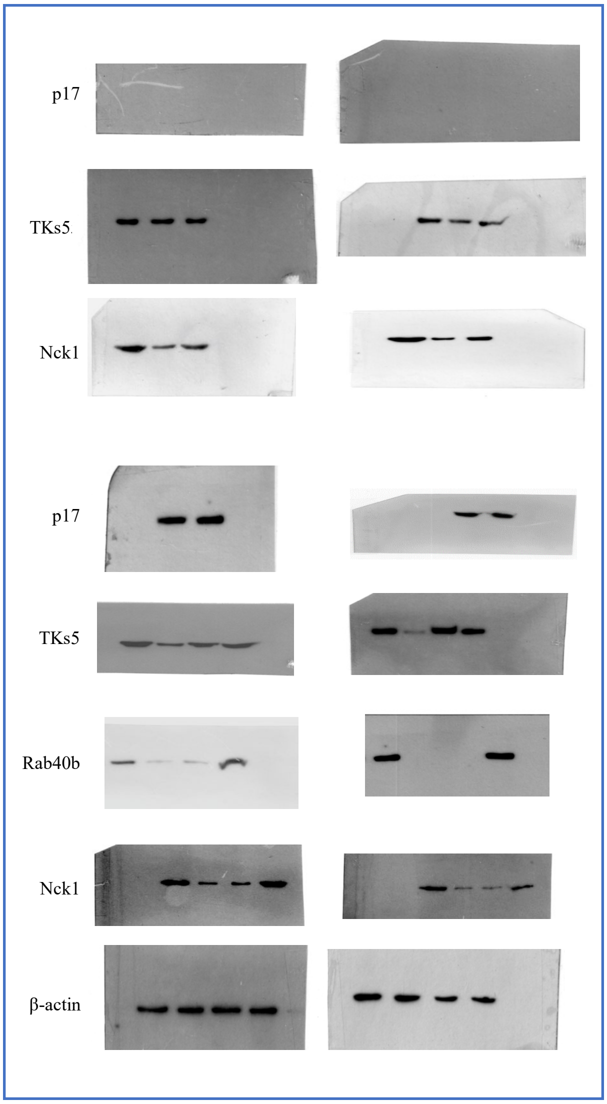

Figure 6B

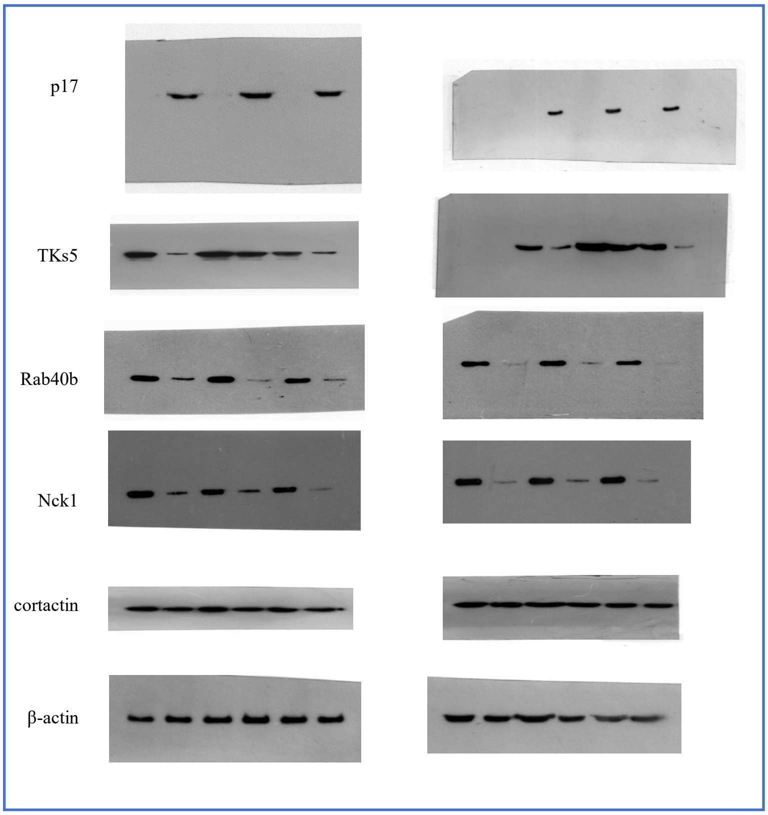

Figure 7

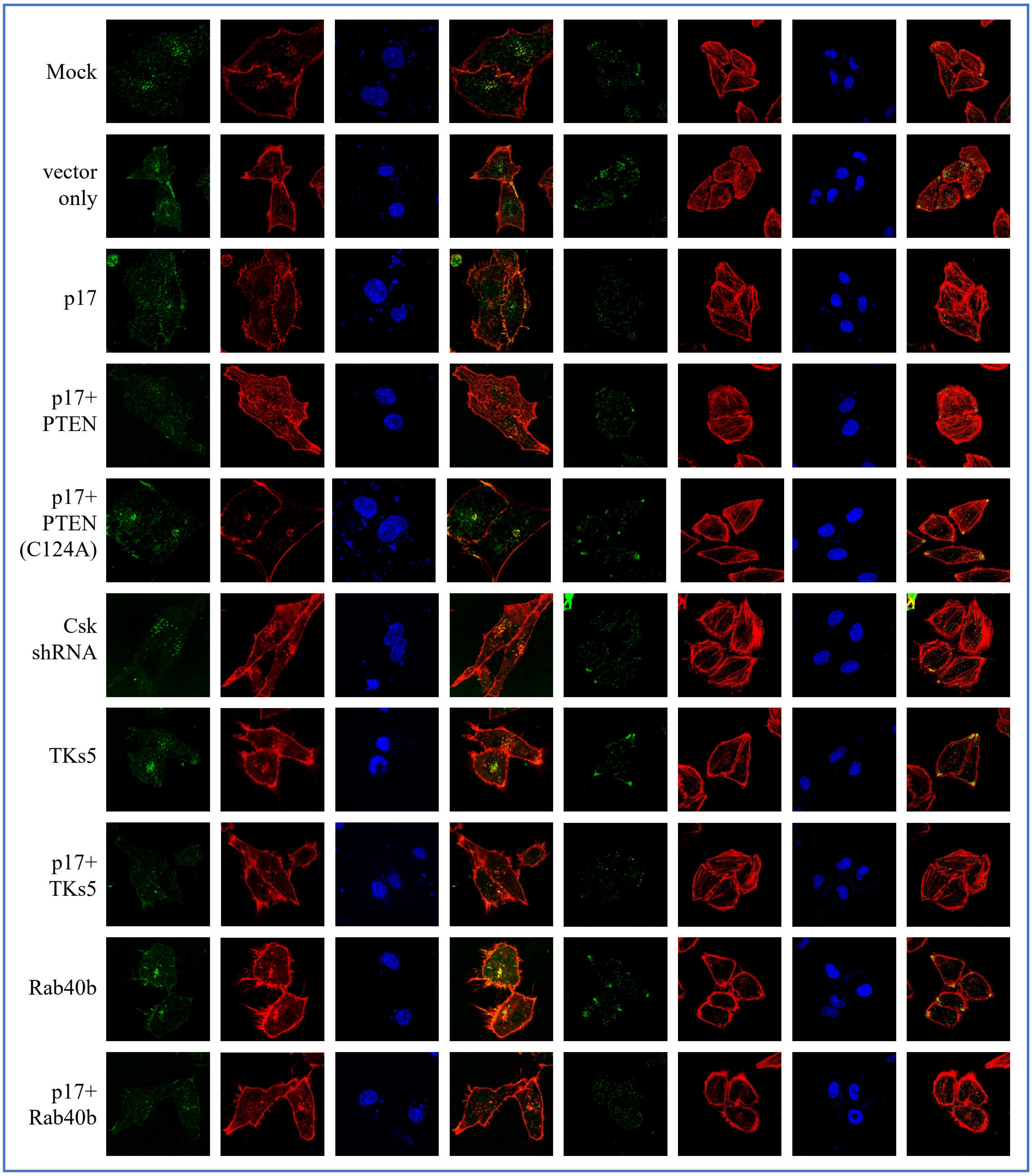

Mock
vector only
p17
p17+ PTEN
p17+ PTEN (C124A)
Csk shRNA
TKs5
p17+ TKs5
Rab40b
p17+ Rab40b

Figure 8

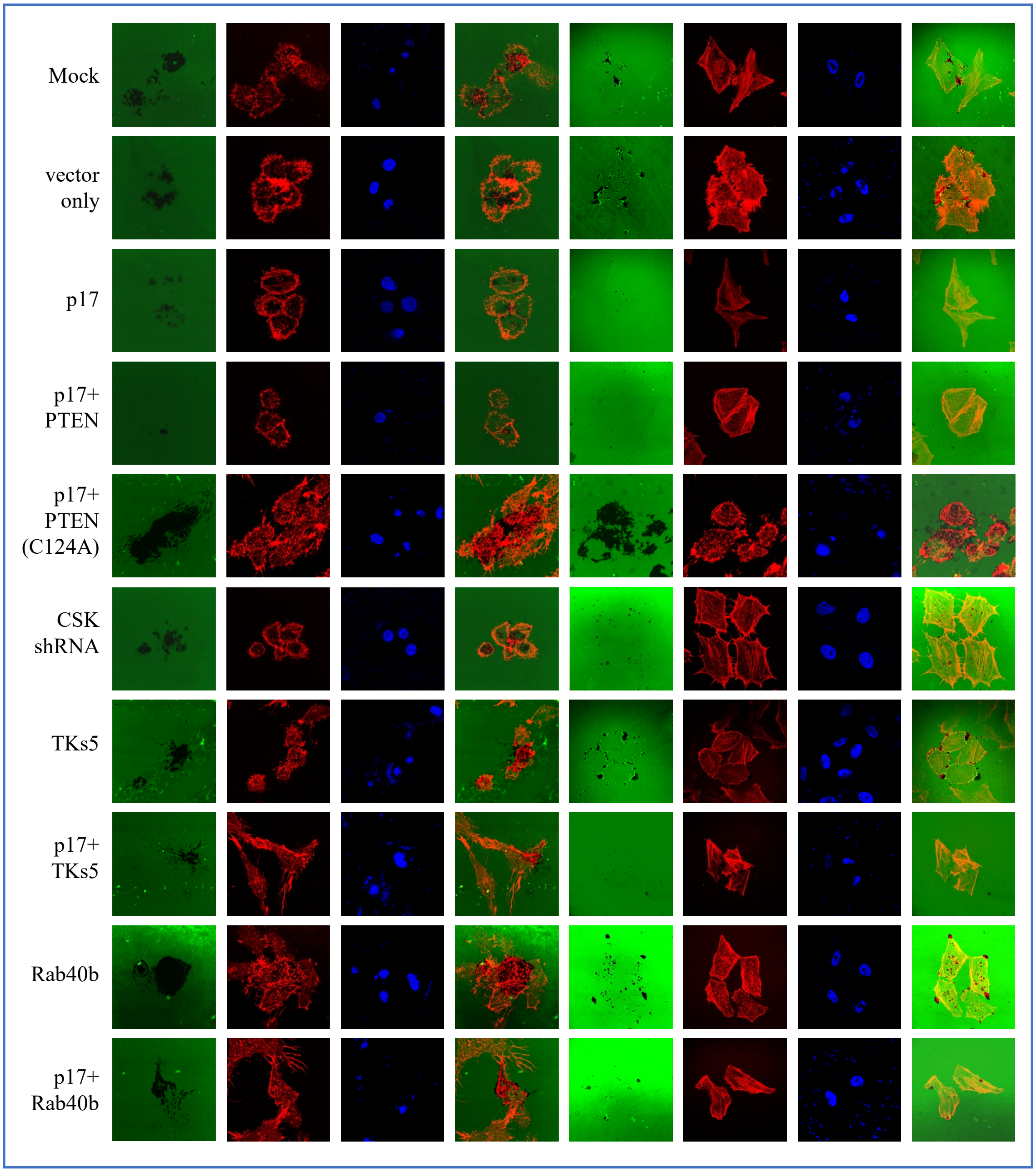

Mock
vector only
p17
p17+ PTEN
p17+ PTEN (C124A)
CSK shRNA
TKs5
p17+ TKs5
Rab40b
p17+ Rab40b

Figure S1A

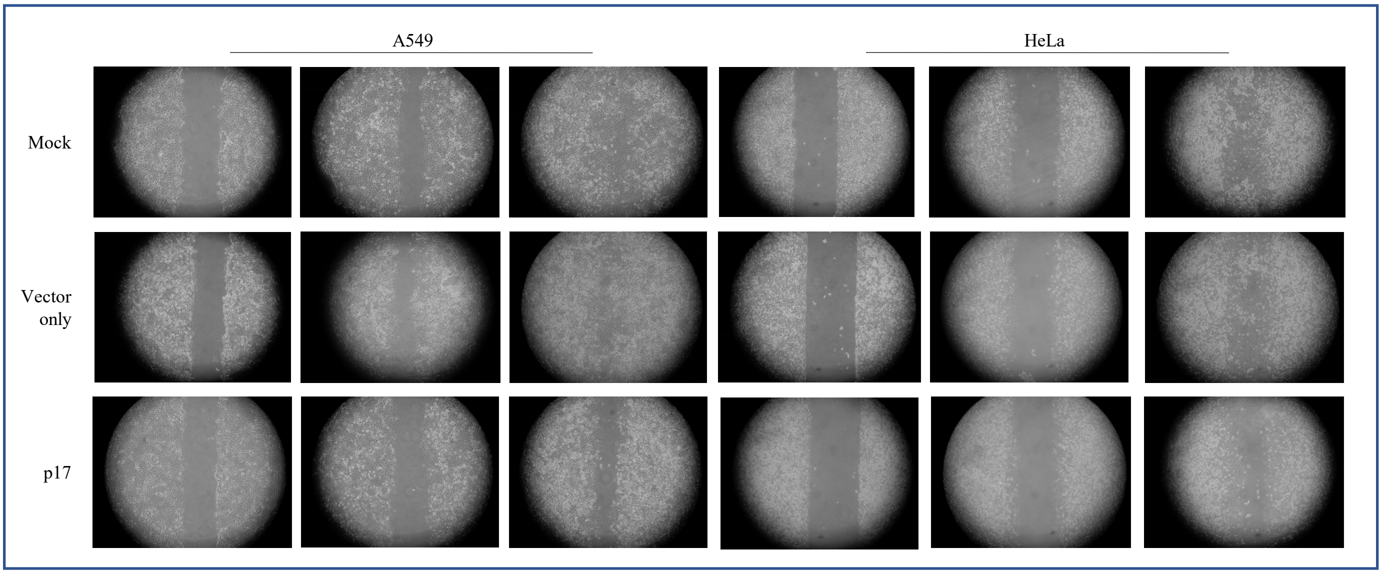

Figure S1B

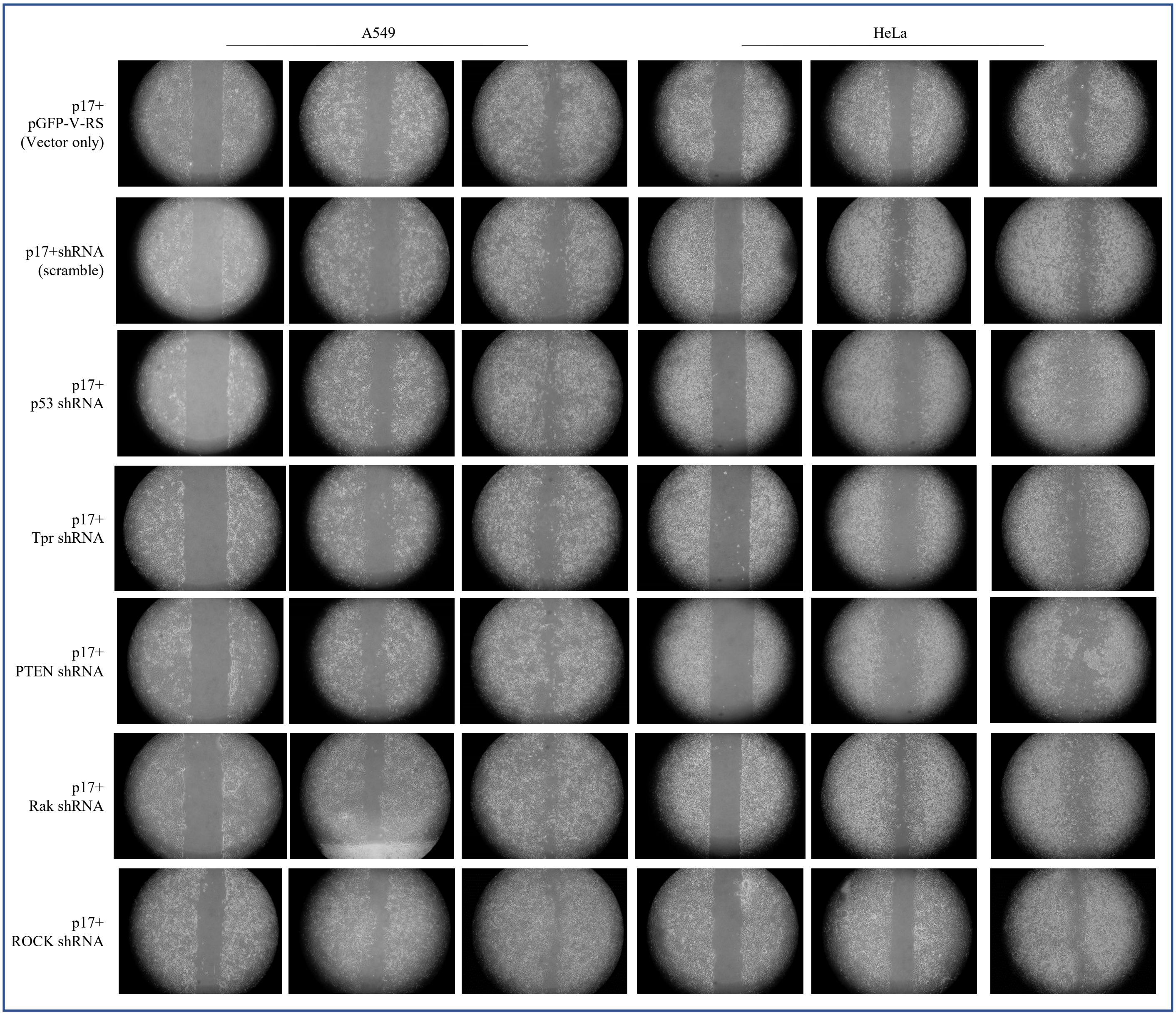

Figure S4 All original blots and images
