# Supplementary material for: The oncolytic avian reovirus p17 protein suppresses invadopodia formation via disruption of TKs5 complexes and oncogenic signaling pathways
Source: Front Cell Infect Microbiol. 2025 Jun 12;15:1603124. doi: 10.3389/fcimb.2025.1603124 (PMC12198159; doi:10.3389/fcimb.2025.1603124)
Supplement: Supplementary file 2 [file DataSheet2.pdf]

Figure S4

Figure 1

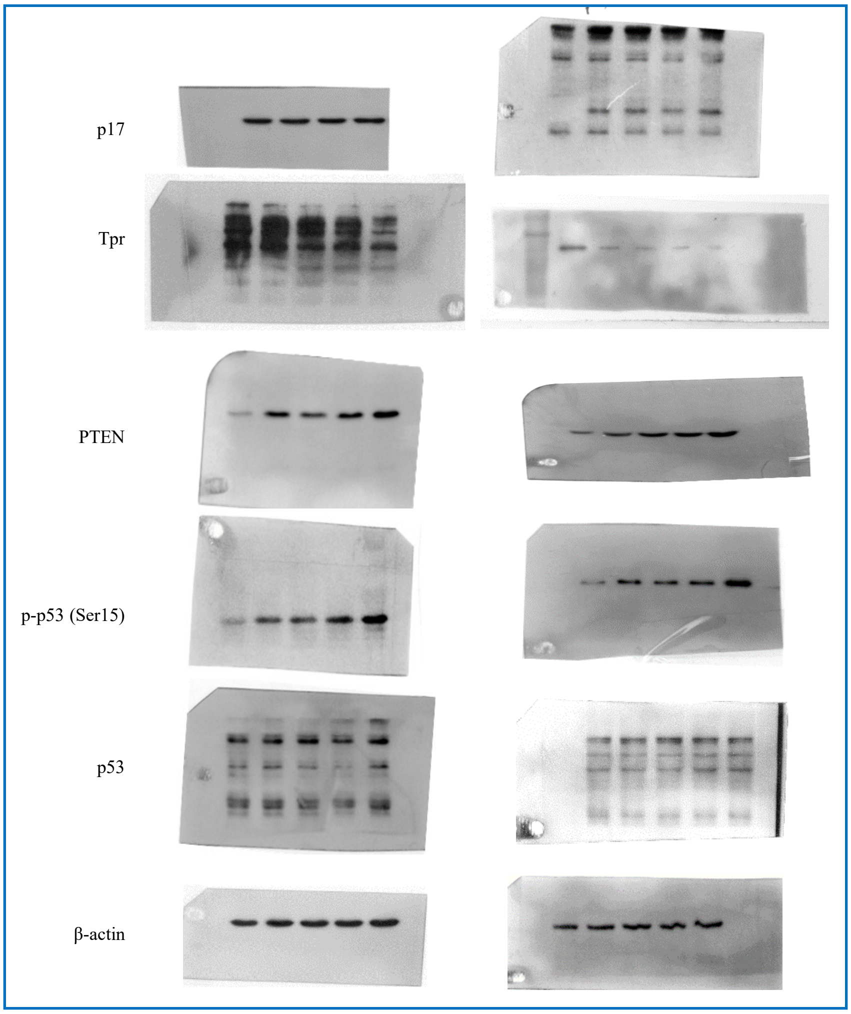

Figure 2

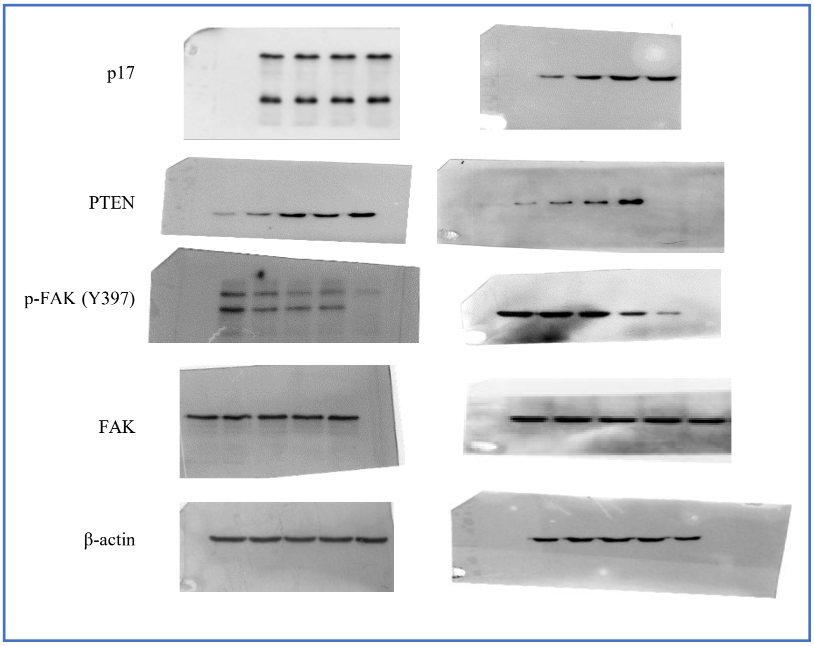

Figure 3

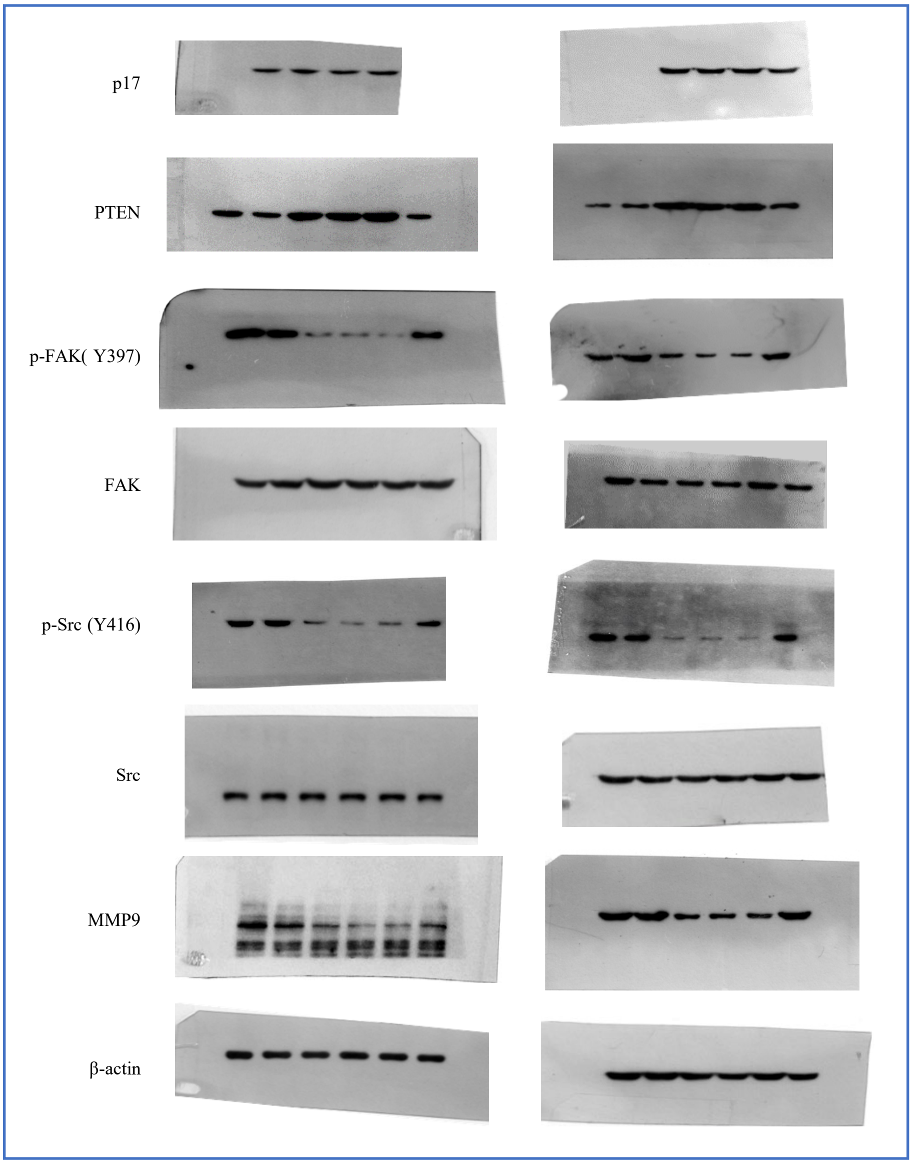

Figure 4

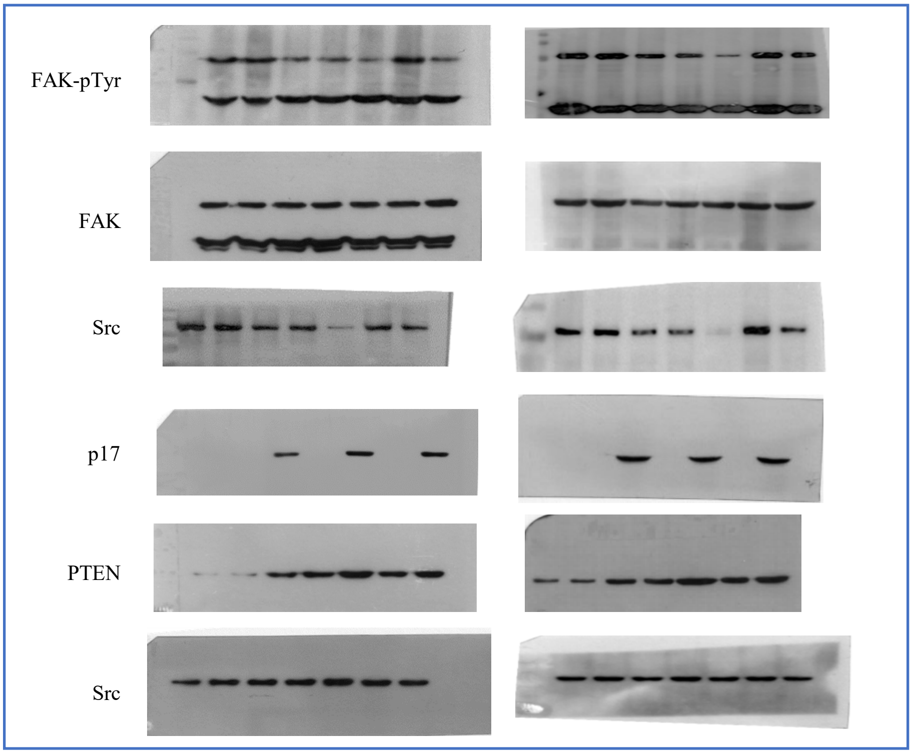

Figure 5A

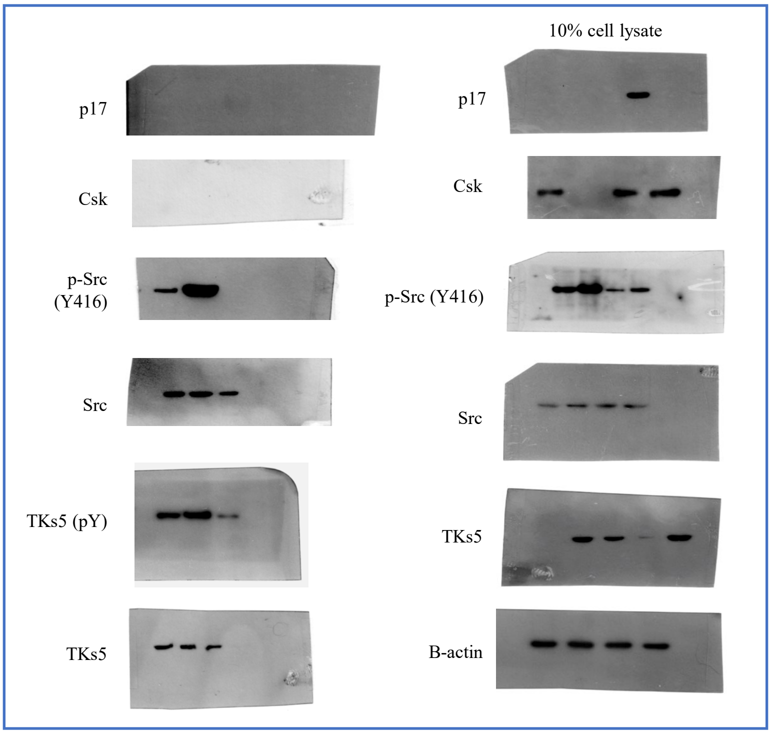

Figure 5B

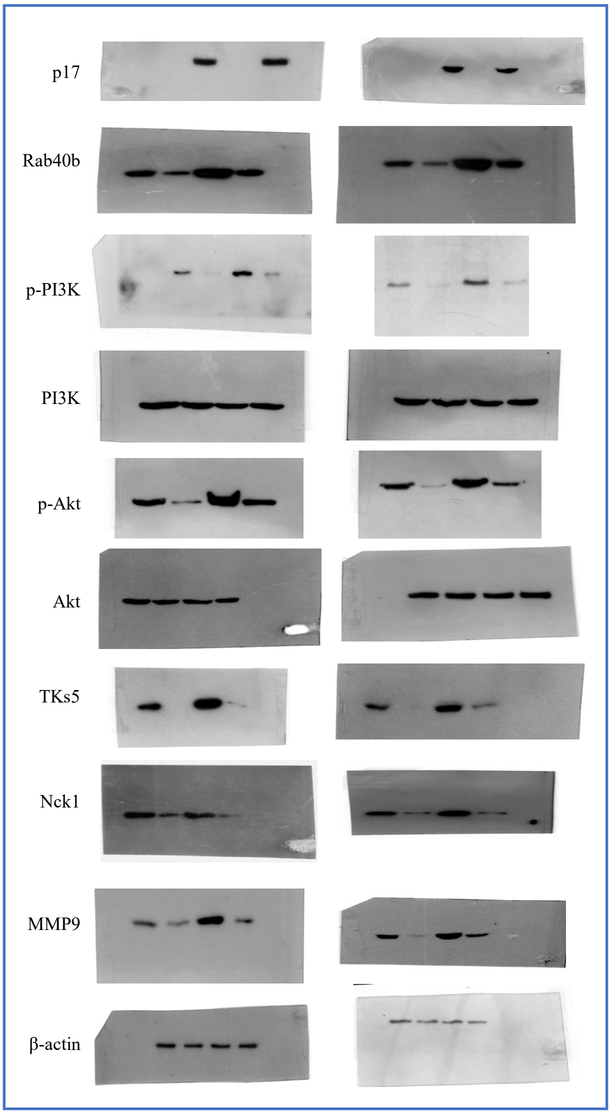

Figure 5C

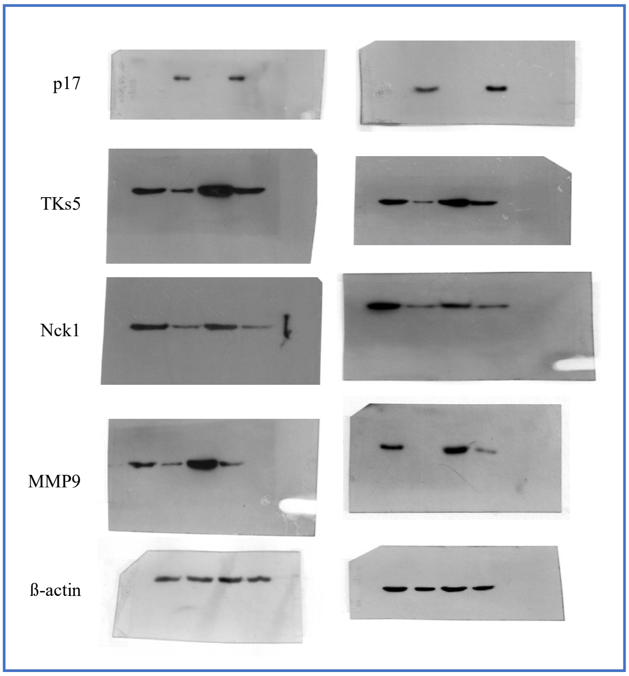

Figure 5D

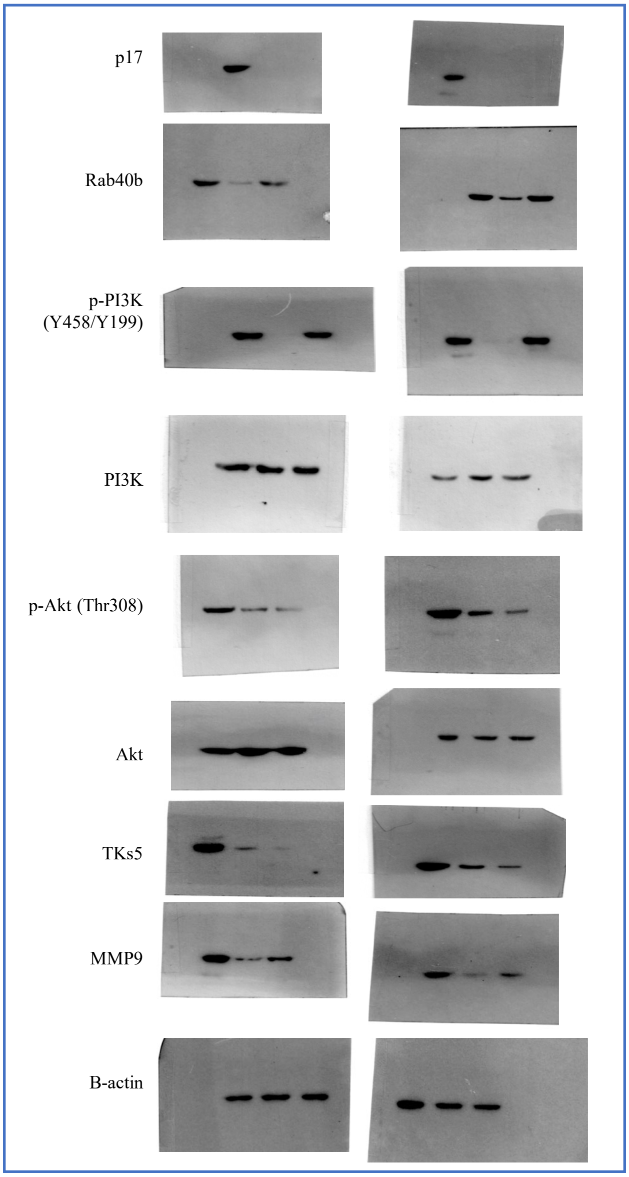

Figure 6A

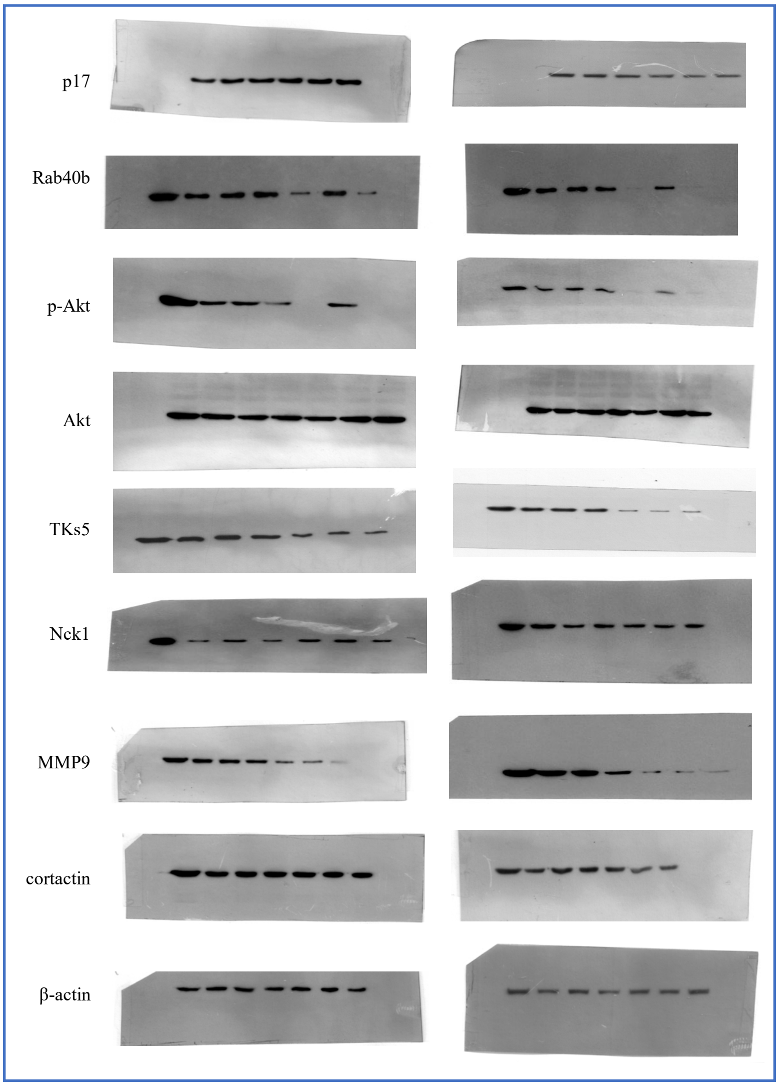

Figure 6B

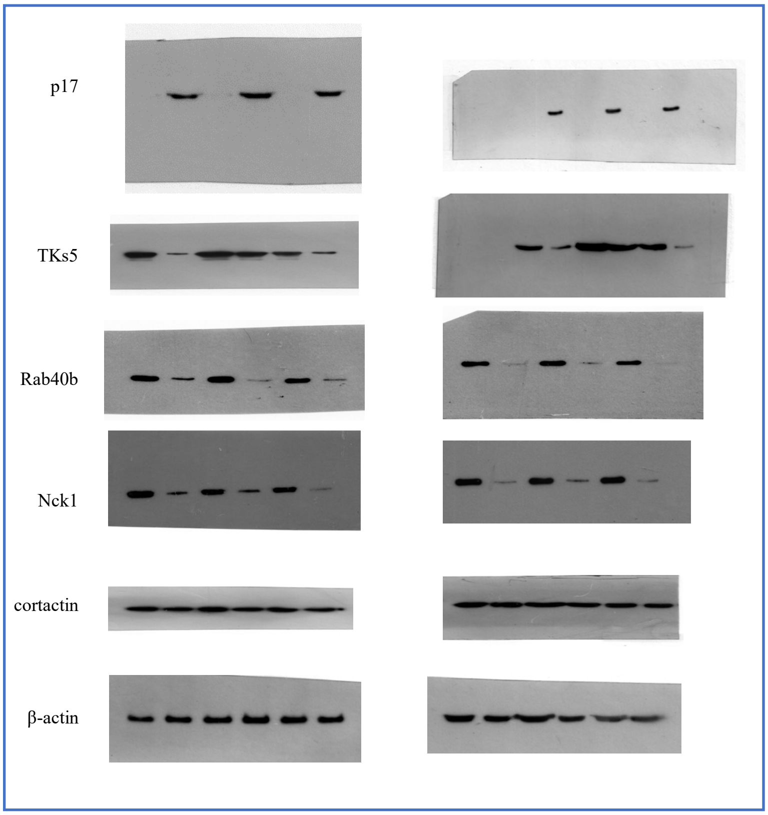

Figure 6C

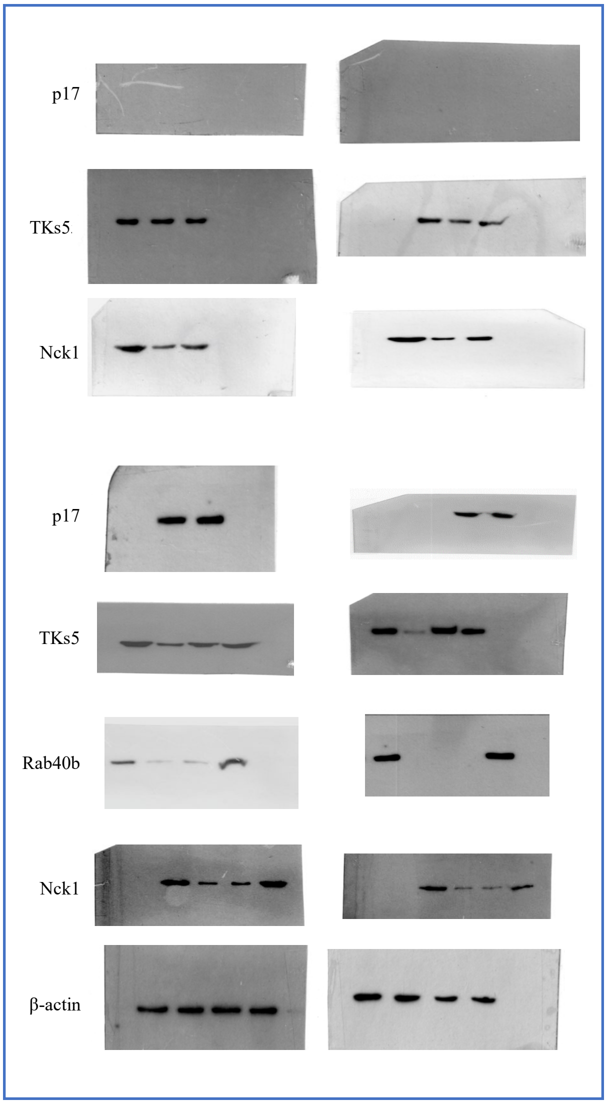

Figure 7

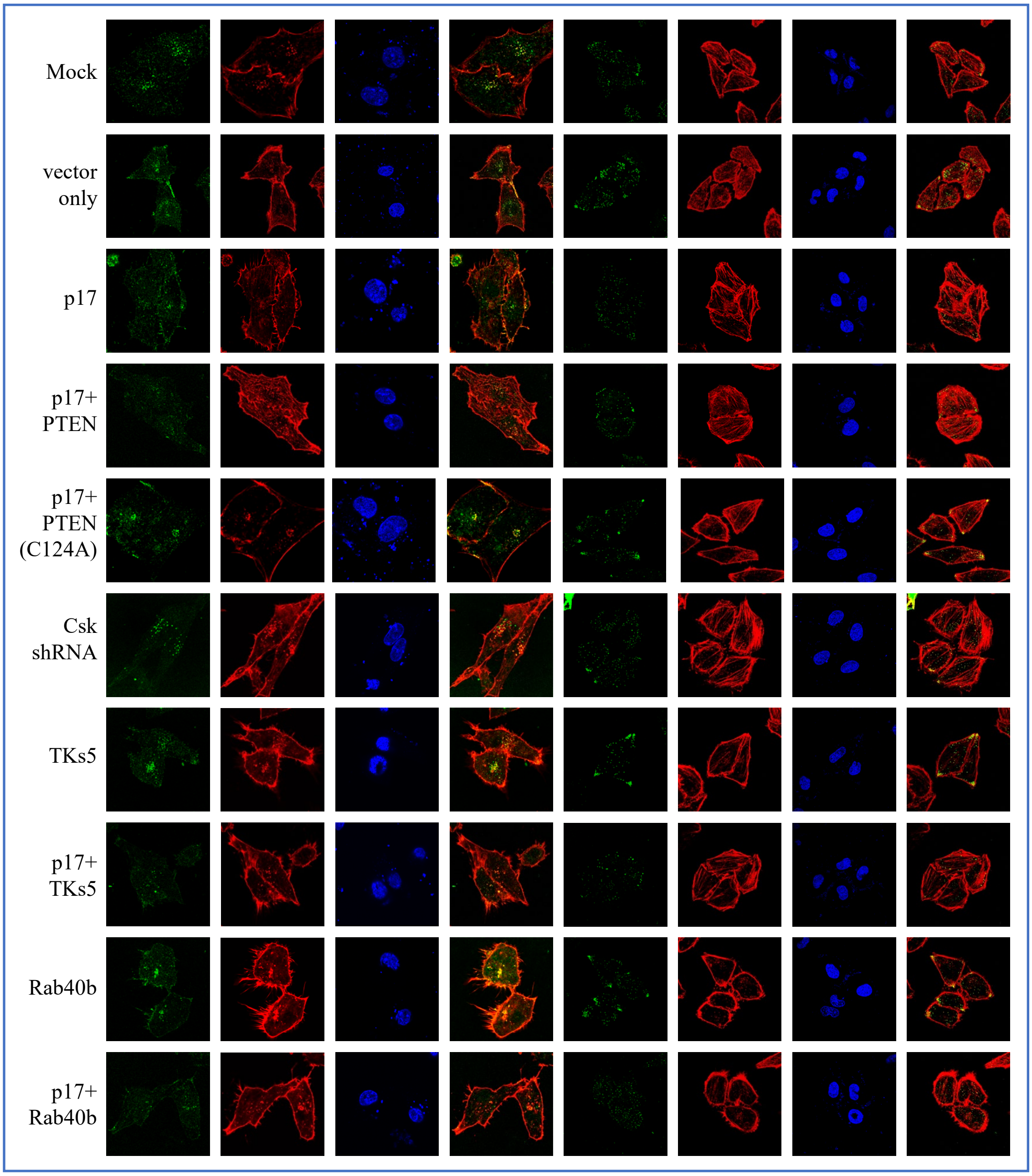
Mock
vector only
p17
p17+ PTEN
p17+ PTEN (C124A)
Csk shRNA
TKs5
p17+ TKs5
Rab40b
p17+ Rab40b

Figure 8

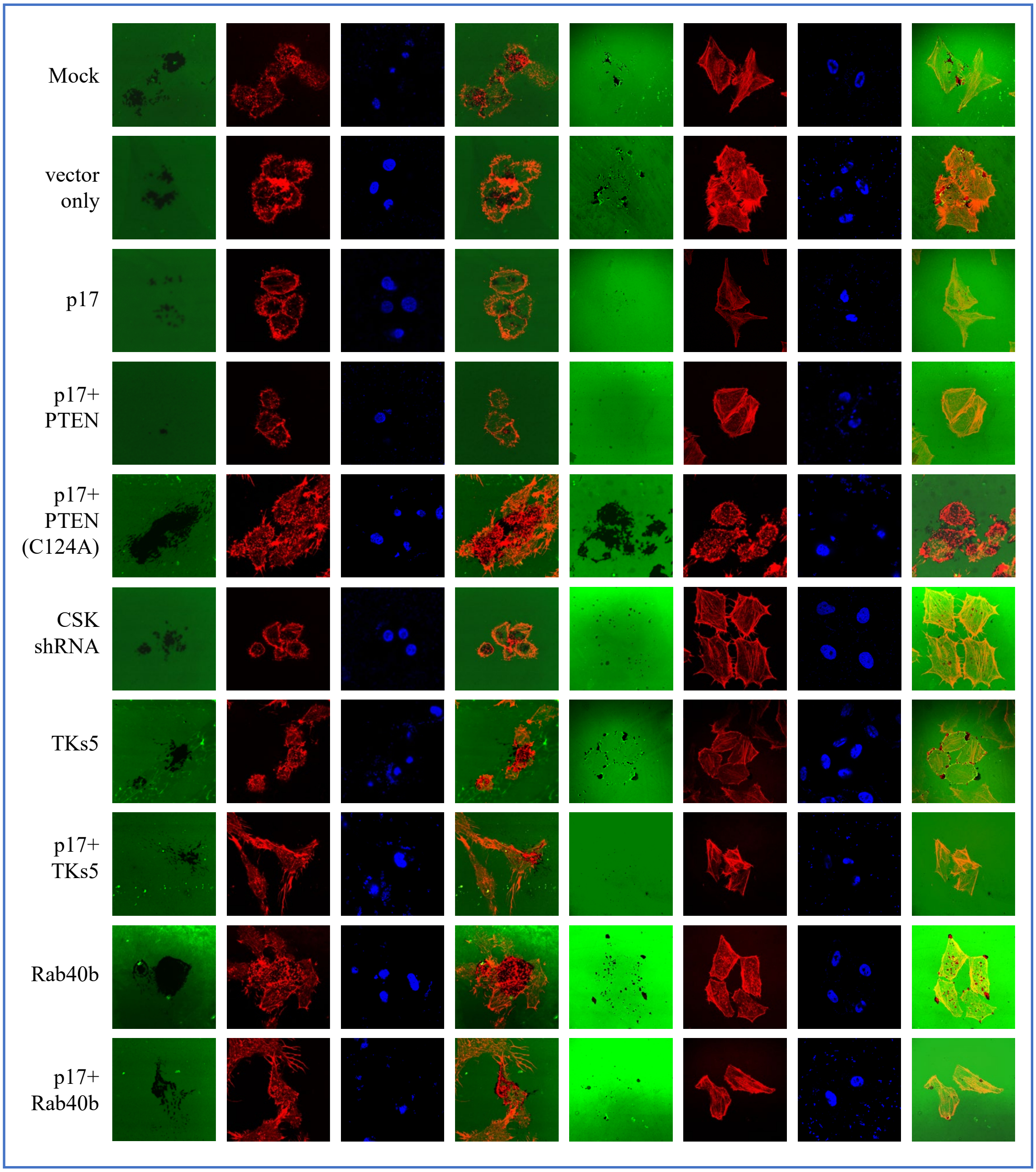
Mock
vector only
p17
p17+ PTEN
p17+ PTEN (C124A)
CSK shRNA
TKs5
p17+ TKs5
Rab40b
p17+ Rab40b

Figure S1A

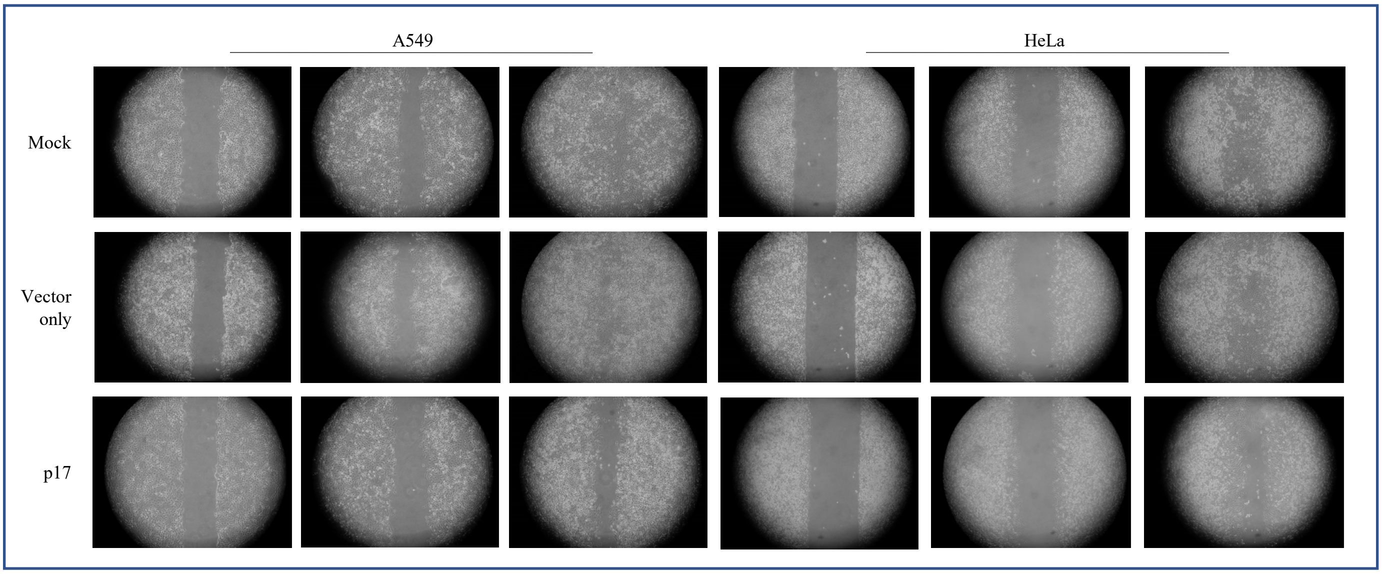

Figure S1B

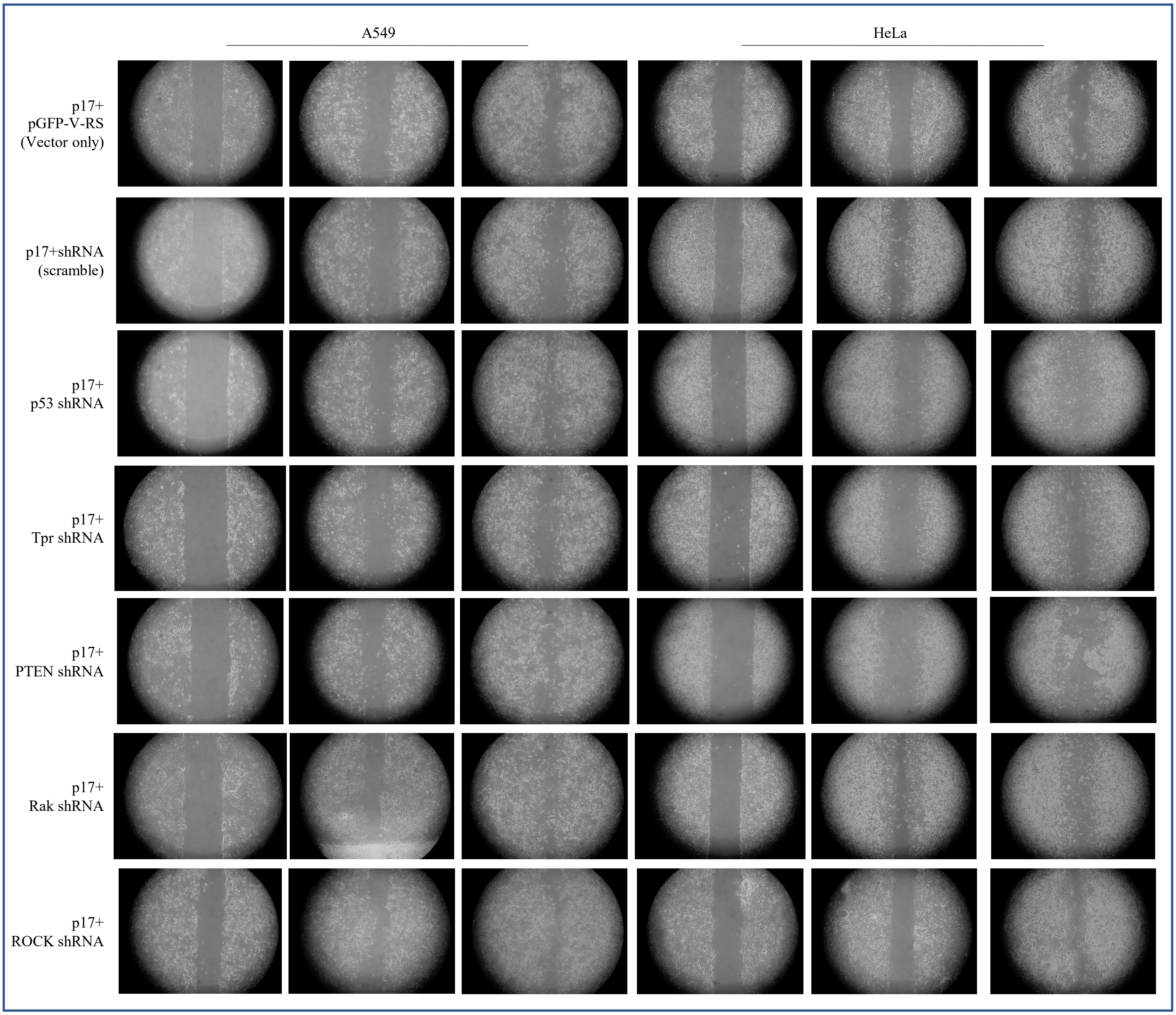

Figure S4 All original blots and images
